# Decoding brain activity using a large-scale probabilistic functional-anatomical atlas of human cognition

**DOI:** 10.1371/journal.pcbi.1005649

**Published:** 2017-10-23

**Authors:** Timothy N. Rubin, Oluwasanmi Koyejo, Krzysztof J. Gorgolewski, Michael N. Jones, Russell A. Poldrack, Tal Yarkoni

**Affiliations:** 1 Department of Psychological and Brain Sciences, Indiana University Bloomington, Bloomington, IN, United States of America; 2 SurveyMonkey, San Mateo, CA, United States of America; 3 Department of Computer Science, University of Illinois at Urbana-Champaign, Urbana, IL, United States of America; 4 Department of Psychology, Stanford University, Stanford, CA, United States of America; 5 Department of Psychology, University of Texas at Austin, Austin, TX, United States of America; Harvard University, UNITED STATES

## Abstract

A central goal of cognitive neuroscience is to decode human brain activity—that is, to infer mental processes from observed patterns of whole-brain activation. Previous decoding efforts have focused on classifying brain activity into a small set of discrete cognitive states. To attain maximal utility, a decoding framework must be open-ended, systematic, and context-sensitive—that is, capable of interpreting numerous brain states, presented in arbitrary combinations, in light of prior information. Here we take steps towards this objective by introducing a probabilistic decoding framework based on a novel topic model—Generalized Correspondence Latent Dirichlet Allocation—that learns latent topics from a database of over 11,000 published fMRI studies. The model produces highly interpretable, spatially-circumscribed topics that enable flexible decoding of whole-brain images. Importantly, the Bayesian nature of the model allows one to “seed” decoder priors with arbitrary images and text—enabling researchers, for the first time, to generate quantitative, context-sensitive interpretations of whole-brain patterns of brain activity.

## Author summary

A central goal of cognitive neuroscience is to decode human brain activity—i.e., to be able to infer mental processes from observed patterns of whole-brain activity. However, existing approaches to brain decoding suffer from a number of important limitations—for example, they often work only in one narrow domain of cognition, and cannot be easily generalized to novel contexts. Here we address such limitations by introducing a simple probabilistic framework based on a novel topic modeling approach. We use our approach to extract a set of highly interpretable latent “topics” from a large meta-analytic database of over 11,000 published fMRI studies. Each topic is associated with a single brain region and a set of semantically coherent cognitive functions. We demonstrate how these topics can be used to automatically “decode” brain activity in an open-ended way, enabling researchers to draw tentative conclusions about mental function on the basis of virtually any pattern of whole-brain activity. We highlight several important features of our framework, notably including the ability to take into account knowledge of the experimental context and/or prior experimenter belief.

## Introduction

A central goal of cognitive neuroscience is to understand how neural and cognitive function interrelate. An important component of this effort is to be able to *decode* cognitive processes from brain activity—that is, to infer mental processes from observed patterns of whole-brain activation—or vice versa. Although researchers have dedicated increasing effort to the challenges of brain decoding [1–4], the vast majority of brain decoding studies to date have focused on fine-grained analysis of a restricted set of cognitive states or experimental tasks—for example, classifying which word or picture a subject is currently perceiving [5,6], or which of several predefined tasks they are engaged in [7,8]. Such work is notable for its ability to achieve high classification rates of very specific stimuli. However, this accuracy is typically purchased at the cost of high context-specificity: thus far, there is little evidence that the patterns learned by classifiers in such studies can capably generalize to new research sites, experimental designs, and subject populations.

By contrast, much less work has focused on the development of open-ended decoding approaches—One approach to this type of generalizable decoding is to use large-scale meta-analytic databases such as Neurosynth [9] and BrainMap [10,11] to derive estimates of what a broad variety of brain activations imply about cognitive processing—a form of analysis widely known as *reverse inference* [12,13]. Such efforts necessarily trade fidelity for breadth; that is, they allow researchers to draw inferences about almost any cognitive process that has been frequently studied with fMRI, but these inference are coarse, and come with a high degree of uncertainty. An illustrative study was conducted by Chang et al. [14], who used the Neurosynth database to "decode" the functional correlates of three distinct right insula clusters. The analytical strategy involved correlating each insula map with dozens of Neurosynth meta-analysis maps and drawing conclusions about function based on differences in relative similarity (e.g., an anterior insula region showed greatest similarity to executive control-related meta-analysis maps; a ventral insula region showed greatest similarity to affect-related maps; etc.). Other studies have used a similar approach to infer the putative functional correlates of whole-brain maps in a variety of other settings [15–17].

More recently, we have generalized this approach and implemented it in the online Neurosynth [9] (http://neurosynth.org) and NeuroVault [65] (http://neurovault.org) platforms. At present, researchers can upload arbitrary whole-brain maps to the NeuroVault repository and instantly decode them against the entire Neurosynth database. This decoding functionality provides researchers with a quantitative means of interpreting whole-brain activity patterns—potentially replacing the qualitative conclusions more commonly drawn in the literature. However, the present approach—which is based entirely on computation of spatial similarity coefficients between the input map and comparison meta-analysis maps—has several weaknesses that limit its utility as a general-purpose decoding framework. Chief among these is that the approach is not grounded in a formal model: it allows one to estimate the similarity of any given brain activity map to other canonical maps, but does not provide a principled way to interpret these mappings. Additionally, it does not attempt to identify any latent structure that presumably makes such mappings useful—for example, individual brain regions or functional brain networks that correspond to specific cognitive processes.

By contrast, a generative framework for decoding brain activity—i.e., one that learns the joint probabilities of all observed and latent variables in the model, and thus can be used to generate new observations—would offer researchers a number of important benefits. First, it would facilitate the learning of interpretable latent structures from a mass of superficial brain-cognition mappings, rather than simply specifying the most likely class (e.g., cognitive task or psychological state) conditional on the observed pattern of activations (as in a discriminative model). Second, a generative model could function bidirectionally, simultaneously supporting both encoding and decoding [18]. That is, in contrast to most decoding models, which predict likely cognitive tasks or mental states on the basis of brain activity, a generative model additionally encodes descriptions of experimental tasks or psychological concepts in image space—enabling researchers to construct hypothetical patterns of brain activity that are consistent with the existing model but may have never been actually observed before (e.g., what pattern of brain activity would a task combining painful stimulation and phonological awareness produce?).

Perhaps most importantly, by virtue of explicitly modeling both the joint and marginal probabilities of all events, a generative framework would provide the ability to contextualize predictions through the explicit use of Bayesian priors. Discriminative brain decoding approaches (which model only the conditional probability of different target states given observed patterns of activity) are inherently acontextual in this sense, and provide no way to integrate contextual information or prior belief into the decoding process. Since many if not most brain regions are generally understood to contain multiple circuits with potentially distinguishable functions, knowledge of the experimental context within which a pattern of brain activity unfolds should, in principle, constrain interpretation of observed brain activity. Left inferior frontal gyrus activation may mean different things in the context of language comprehension [19], emotion regulation [20], or response inhibition [21]. More generally, true reverse inference—i.e., the move to draw conclusions about the likelihood of different mental states conditional on observed brain activity—is an inherently Bayesian notion that requires one to formally model (and specify) the prior probability of each term or concept's occurrence. Whereas a similarity-based decoding approach cannot easily support such specification, it is intrinsic to a generative model.

Here we take the first steps towards these goals by introducing an unsupervised generative Bayesian decoding framework based on a novel topic model—Generalized Correspondence Latent Dirichlet Allocation (GC-LDA)—that learns latent topics from the meta-analytic Neurosynth database of over 11,000 published fMRI studies [9]. GC-LDA generates topics that are simultaneously constrained by both anatomical and functional considerations: each topic defines a spatial region in the brain that is associated with a highly interpretable, coherent set of cognitive terms. In principle, this joint estimation approach should produce more parsimonious brain-cognition mappings than the more common strategy of factorizing brain activation data by itself and then attempting to project the resulting components onto cognitive dimensions [14,15,17,22].

We demonstrate that the dictionary of topics produced by the GC-LDA model successfully captures known anatomical and functional distinctions and provides a novel data-driven metric of hemispheric specialization. We then take advantage of the topic model's joint spatial and semantic constraints to develop a bidirectional, open-ended decoding framework. That is, we demonstrate the ability to extract both a text-based representation of any whole-brain image, and a whole-brain activity pattern corresponding to arbitrary text. Importantly, the Bayesian nature of the model allows us to formally specify a decoder's priors by "seeding" it with any arbitrary combination of images and text. The direct consequence is that, for the first time, researchers are able to generative quantitative, context-sensitive interpretations of whole-brain patterns of brain activity.

## Results

### Mapping the functional neuroanatomy of the brain with topic models

Our decoding framework is built on a widely-used Bayesian modeling approach known as *topic modeling* [23,24]. Topic modeling is a dimensionality-reduction technique, which decomposes a corpus of documents into a set of semantically coherent probability distributions over words, known as *topics*. Given this set of topics, each document can be represented as a probabilistic mixture of topics. Topic models have been successfully applied to a wide range of problems, including text classification [25,26], information retrieval [27], image classification [28], and theme discovery [29,30], and are now regarded as a standard technique for text and image analysis. An important feature from a decoding standpoint is that topic models are generative in nature: they allow a principled approach for bidirectional mapping from documents to latent components and vice versa; probabilistic generation of entirely new (i.e., previously unseen) documents; and formal Bayesian updating that can allow for explicit specification of the prior topic probabilities. We return to these features later.

**Fig 1:**
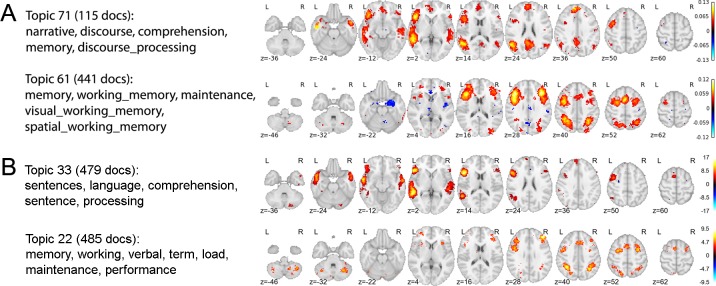
Replication of topics from Poldrack et al. [31]. Figure shows the results of applying the generic LDA model [23] to the Neurosynth database, as described in Poldrack et al. (2012). (A) Selected topics reported in Poldrack et al.[31] using an older Neurosynth database of 5,809 studies. (B) Closest matching topics when applying the same approach to the current, expanded, Neurosynth database (11,406 studies).

In previous work, we used a standard topic model to extract 200 semantically coherent topics from the abstracts of all published fMRI articles contained in an older and smaller version of the Neurosynth database [5,809 studies; 31]. We then projected each topic onto the space of brain activity to identify brain regions associated with distinct cognitive profiles. A direct replication of this earlier approach using the current, and much larger, Neurosynth database (11,406 studies) produces very similar results (e.g., Fig 1). As Fig 1 illustrates, the structure-function mappings produced by this approach converge closely with numerous other findings in the literature—e.g., the presence of a strongly left-lateralized language network [19] and the involvement of dorsal frontoparietal regions in working memory and executive control [32]. However, because the standard topic model operates only on the text of publications, the topics it produces are not constrained in any way by neural data. Furthermore, the spatial mappings for each topic are indirectly computed via the documents’ topic loadings—the spatial data is not built into the model. The result is a set of widely distributed, network-like activation maps that closely resemble the whole-brain maps produced by individual fMRI experiments. While such an approach is informative if one’s goal is to identify the distributed neural correlates of coherent psychological topics, it is of little help in the search for relatively simple, well-defined functional-anatomical atoms. A similar limitation applies to more recent work by Yeo et al, who used a more sophisticated topic model to derive a set of *cognitive components* that map in a many-to-many fashion onto both behavioral tasks and patterns of brain activity [33]. While the latter approach represents an important advance in its simultaneous use of both behavioral and brain activity data, the resulting spatial components remain relatively widely distributed, and do not provide insight into the likely cognitive roles of well-localized brain regions.

### The GC-LDA model

**Fig 2:**
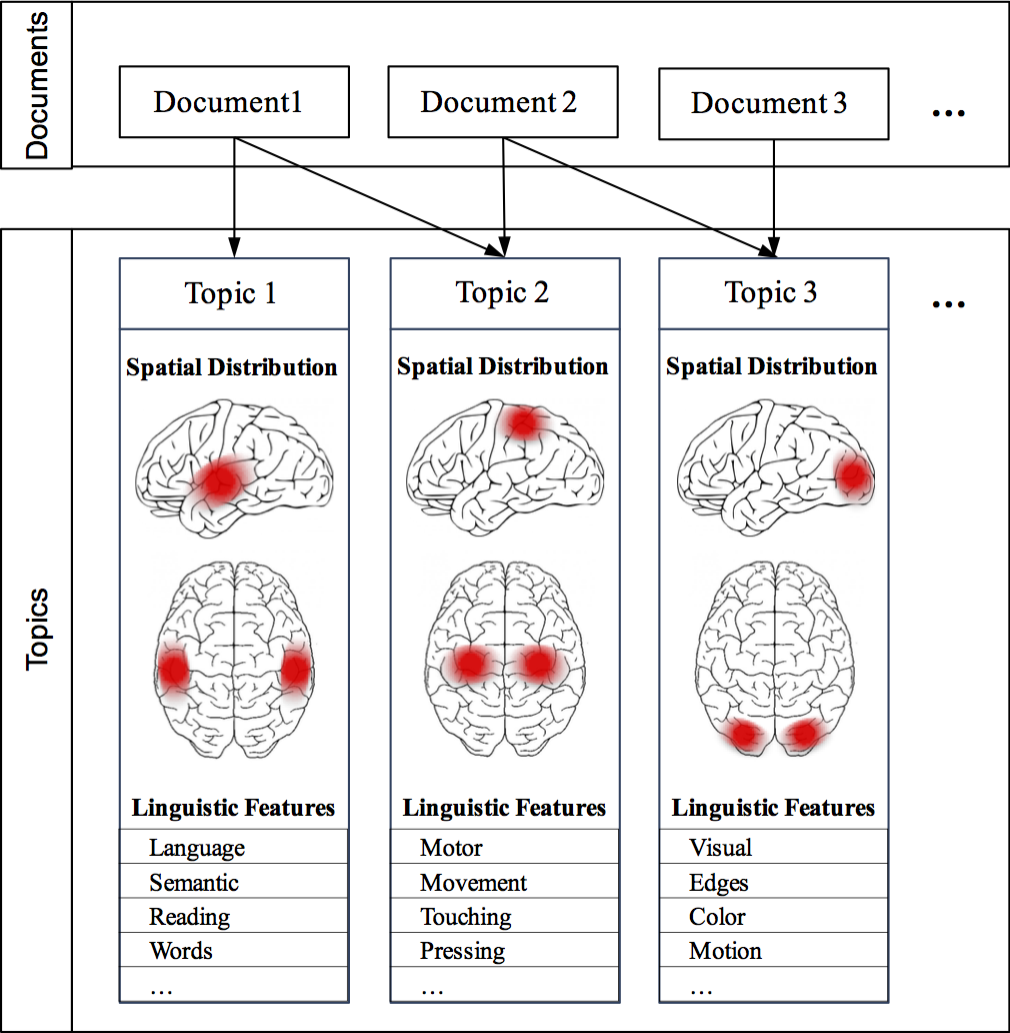
Schematic overview of the GC-LDA model. Each document (an article in the Neurosynth corpus) is represented as a mixture of learned latent topics, where each topic is associated with both a 3-dimensional Gaussian spatial distribution, and a set of linguistic terms extracted from the abstract text.

To extract structure-to-function mappings focused on a more granular, region-like level of analysis, we developed a novel topic model based on the Correspondence-LDA model [34] that generates topics simultaneously constrained by both semantic and spatial information. We term this the Generalized Correspondence LDA (GC-LDA) model [Fig 2; for details, see 35]. The GC-LDA model learns a set of latent topics, each associated with (i) a spatial probability distribution over brain activations and (ii) a probability distribution over words that tend to co-occur in article abstracts. In this context, we use the term *document* to refer to a single Neurosynth article (containing both a list of cognitive terms that occur in the abstract, and a set of reported brain activations).

This extension of the Correspondence-LDA model allows for any spatial distribution to be associated with topics, where the choice of spatial distribution can be made according to the goals of the experimenter. In [33], we considered three variants of the GC-LDA model, where each topic was associated with either: (1) a single multivariate Gaussian distribution, (2) a mixture of two unconstrained Gaussian distributions, or (3) a mixture of two Gaussian distributions that were constrained to be symmetric around the x-axis, such that these regions capture bilateral symmetry. Based on our results in [33] we found that models using a mixture of Gaussians outperformed models using a single Gaussian in terms of their ability to predict held-out data. Furthermore, the symmetrically constrained mixture model enabled us to directly quantify the degree of hemispheric symmetry (or lack thereof) displayed by each topic. For these reasons, we focus in this paper on the model that uses a symmetrically constrained mixture of two Gaussians. We note, however, that this choice of spatial distribution is not "correct" in any normative sense, and simply reflects a pragmatic choice we make for purposes of both interpretability and predictive validity (see [33] for additional discussion).

**Fig 3:**
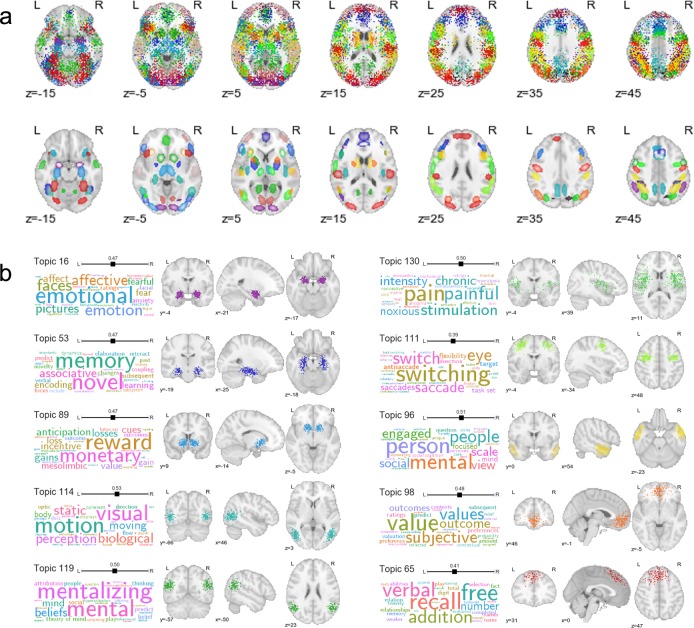
Selected topics learned by the GC-LDA model (for full results, see S1 Fig). (a) Spatial distributions for 90 of the 200 topics. Each color represents a different topic. Top row: hard assignments of activations to topics; each point represents a single activation from a single study in the Neurosynth database (note that each topic is spatially represented by a mixture of only two symmetrically-constrained gaussians; the appearance of multiple regions that share colors is due to the inevitable reuse of perceptually similar colors). Bottom row: estimated multivariate Gaussian mixture distribution of each topic. (b) Top semantic associates (word clouds) and activation distributions (orthogonal brain slices) for selected topics. The size of a term in each word cloud is proportional to the strength of loading on the corresponding topic.

Fig 3 displays selected topics extracted using the GC-LDA model (for comprehensive results, see S1 Fig and neurovault.org/collections/EBAYVDBZ/). As illustrated, the model produced numerous topics that had well-defined joint spatial and semantic representations (Fig 3A)—approximately half of the 200 extracted topics were clearly interpretable (see S1 Fig for full details). Many of these topics successfully captured relatively basic associations between specific structures and their putative functions; for example, we identified topics associated with amygdala activation and emotion; reward and the ventral striatum; hippocampus and memory; fusiform face area and face perception; and motion perception and the V5/MT complex, among others (Fig 3B). In other cases, the model successfully captured and localized higher-level cognitive processes—e.g., topics associated with the temporoparietal junction and mentalizing, temporal pole and person perception, or ventromedial PFC and valuation, among others (Fig 3B). In supplementary analyses, we further demonstrate that the full set of 200 topics can be used to accurately “reconstruct” arbitrary patterns of whole-brain activity, providing an interpretable, low-dimensional way to summarize virtually any whole-brain image (Supporting Results).

### Probabilistic structure-to-function mapping

**Fig 4:**
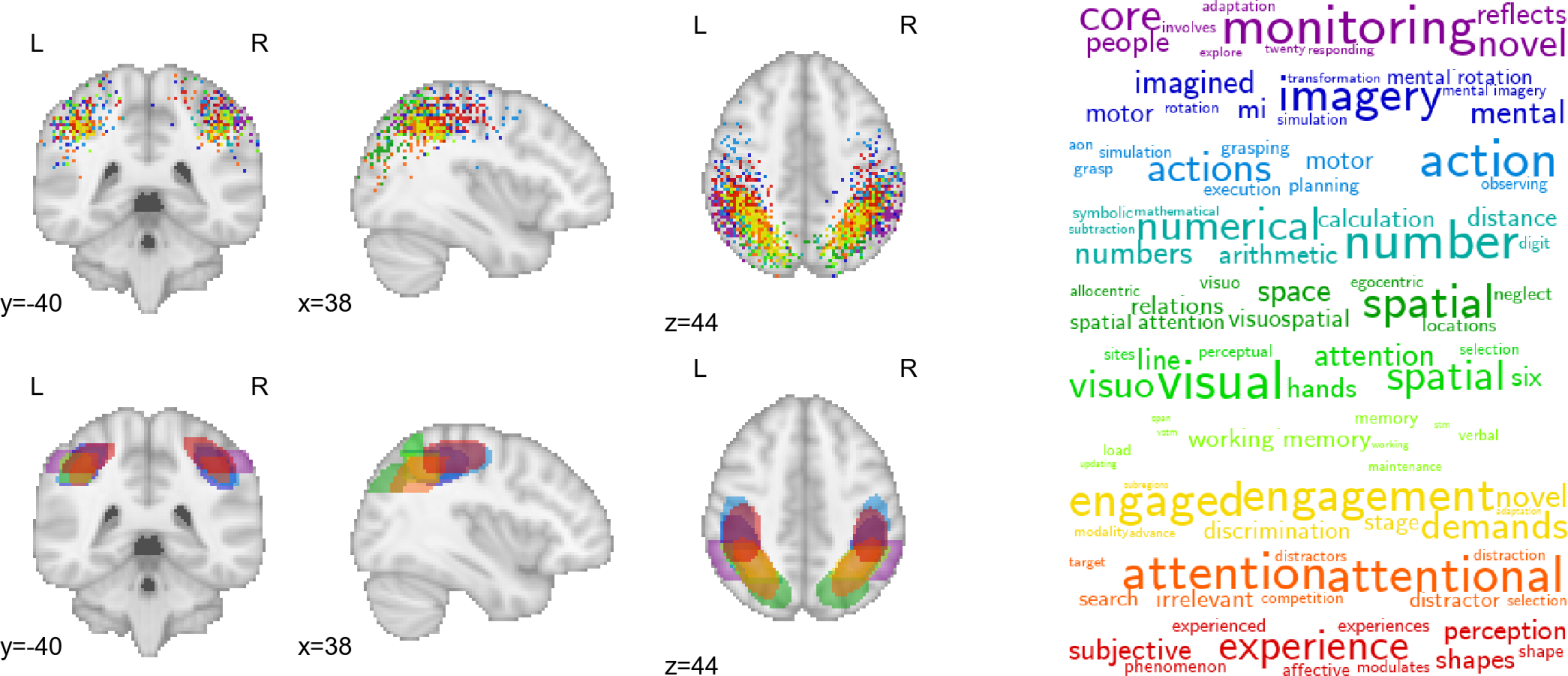
Activation profiles and top-loading words for spatially overlapping topics in parietal cortex. Top row: hard assignments of activations to topics; each point represents a single activation from a single study in the Neurosynth database. Bottom row: estimated multivariate Gaussian mixture distribution of each topic.

An important feature of the GC-LDA model is that it avoids the common, but restrictive, clustering assumption that each voxel should only be assigned to a single group [36–40]. By allowing extracted topics to overlap with one another in space, the model explicitly acknowledges that the brain contains spatially overlapping circuits with thematically related functions. Fig 4 illustrates the close spatial and semantic relationships between 10 different topics localized to overlapping parts of the parietal cortex along the banks of the intraparietal sulcus (IPS). Note the particularly similar posterior parietal cortex (PPC) distributions of topics associated with visuospatial processing, working memory, and general task engagement. These results are consistent with electrophysiological findings of highly heterogeneous, and typically complex, response profiles in PPC neurons [41–43, including coding of visual object location, direction of attention, motor plans, etc.; 44], and underscore the difficulty individual fMRI studies may face in trying to isolate brain-cognition mappings via a hemodynamic signal that sums over millions of neurons at each voxel.

**Fig 5:**
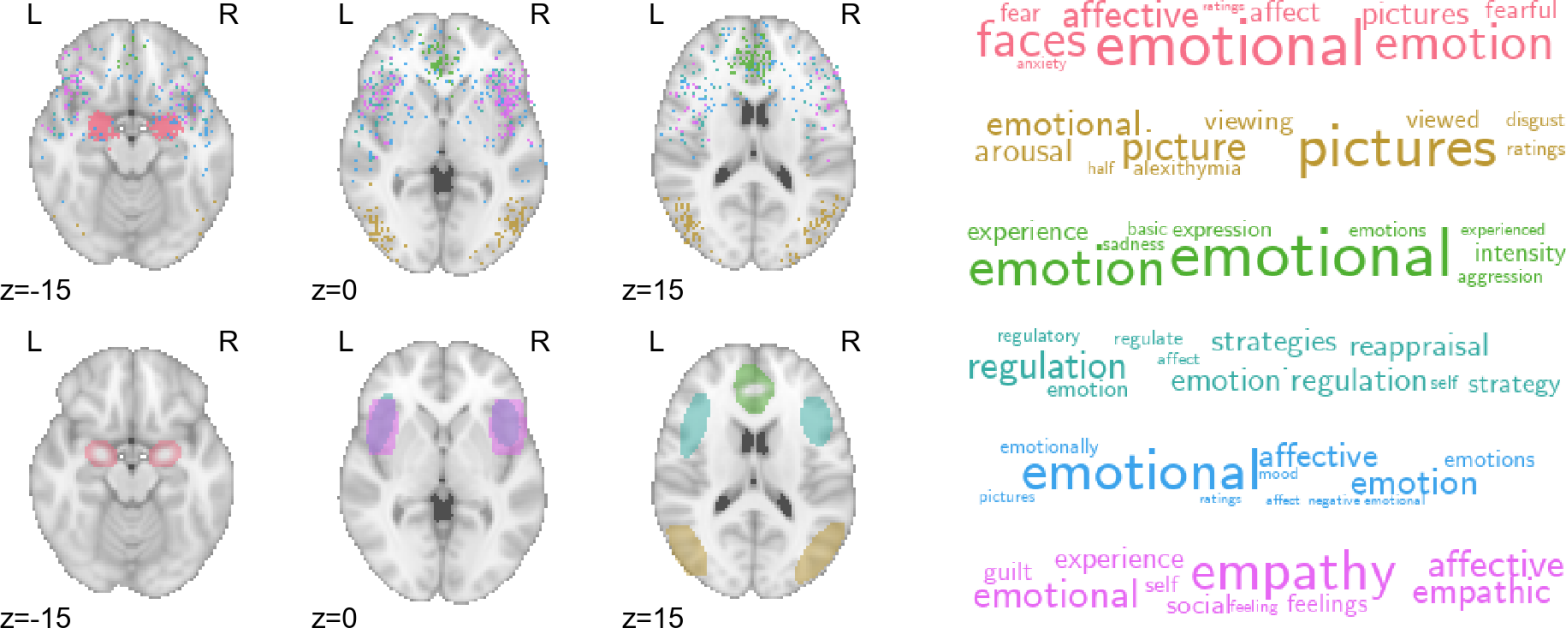
Activation profiles and top-loading words for emotion-related topics. Top row: hard assignments of activations to topics; each point represents a single activation from a single study in the Neurosynth database. Bottom row: estimated multivariate Gaussian mixture distribution of each topic.

Analogously, the probabilistic nature of the GC-LDA mappings can also provide insights into the compositional character of most cognitive states—i.e., the fact that most states are likely to recruit activation of a number of spatially distinct brain regions. Fig 5 displays activation and word distributions for a number of emotion-related topics. Different topics captured different aspects of emotional processing: consistent with extensive previous work, extrastriate visual cortex and amygdala were associated with perceptual processing of emotion [45–47]; rostral anterior cingulate cortex and anterior insula were associated with experiential aspects of emotion [48]; and lateral frontal cortex was associated with emotion regulation [20,49].

### A data-driven window into lateralization of function

**Fig 6:**
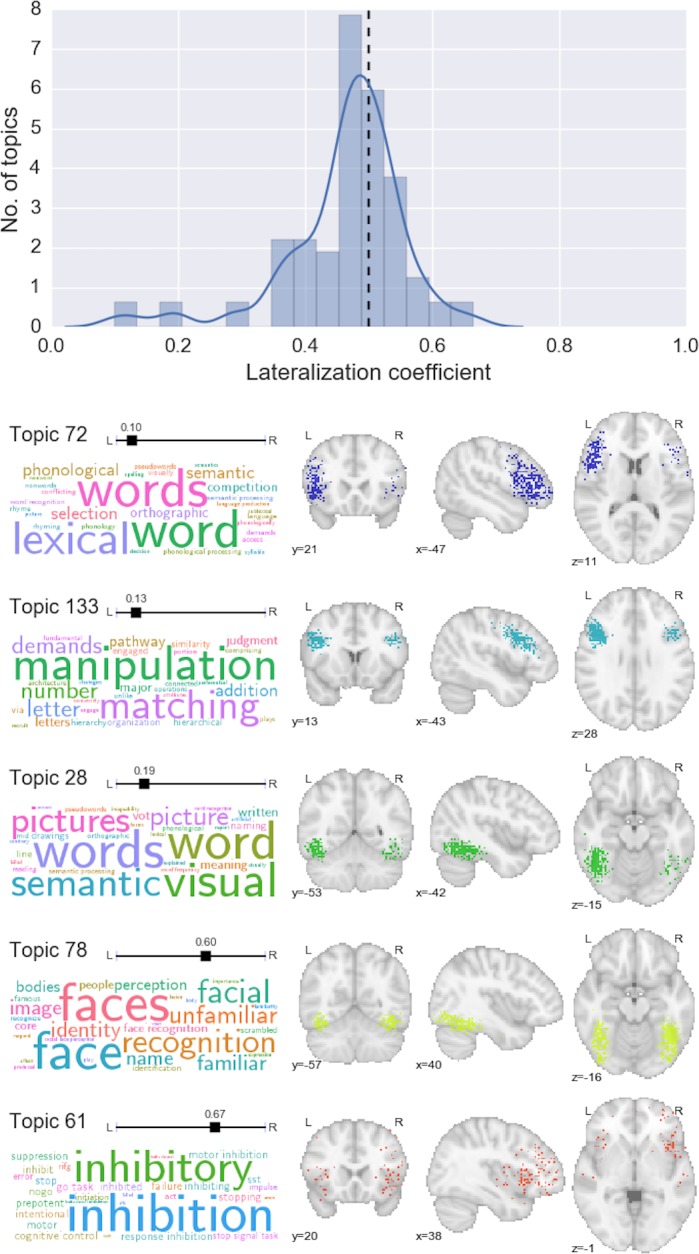
Data-driven estimation of hemispheric lateralization of cognitive function. Top: histogram and kernel density estimation plot of the lateralization coefficient for all topics. Values below 0.5 represent left-lateralization; values above 0.5 represent right-lateralization. Bottom: selected topics that displayed notable hemispheric lateralization.

As noted above, each topic in the GC-LDA model was deliberately constrained to reflect two sub-regions reflected around the brain’s x-axis. This constraint allowed us to estimate the relative weight of activations for each topic in the left vs. right hemisphere—in effect providing a novel, data-driven index of hemispheric specialization. As one might expect given the marked degree of activation symmetry observed in most fMRI studies, most topics showed little or no hemispheric bias (Fig 6, top). However, there were a number of notable exceptions (e.g., Fig 6, bottom). Several language-related topics localized strongly to left-hemisphere language regions—including inferior and middle frontal gyrus, posterior superior temporal sulcus, and inferotemporal cortex [encompassing the putative visual word form area; 50]. Right-lateralized topics were fewer in number and generally showed a weaker hemispheric asymmetry, but notably included a face processing topic localized to the putative fusiform face area [51], and an inhibitory control-related topic localized to the right ventral anterior insula [52]. To our knowledge, these findings constitute the first data-driven estimation of region-level functional hemispheric asymmetry across the whole brain.

### Automatic text-to-image and image-to-text decoding

**Fig 7:**
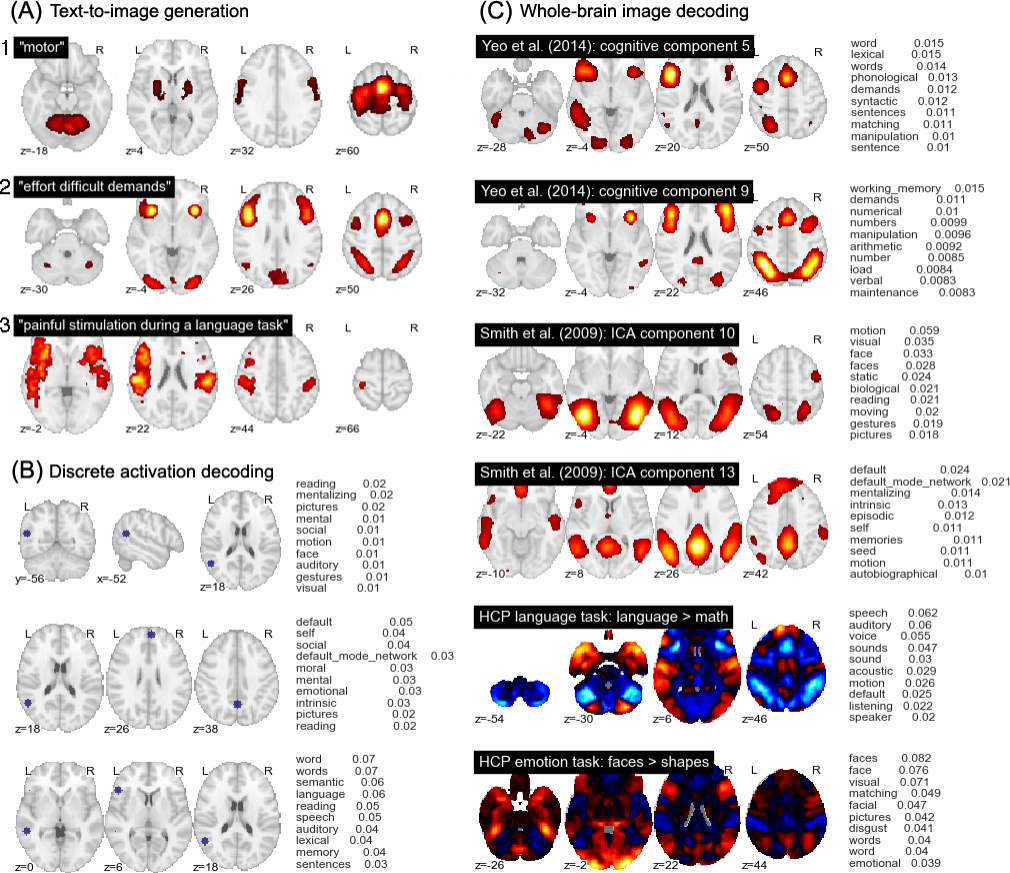
Examples of generative text-to-image and image-to-text mapping using the trained GC-LDA model. (A) Generation of predicted whole-brain images from arbitrary text. (B) Topic-based decoding of discrete activation coordinates. (C) Topic-based decoding of continuous whole-brain images; examples selected from the cognitive components reported in Yeo et al. [33], the BrainMap ICA components reported in Smith et al. [15], and the language and emotion contrasts from the n = 500 release of the HCP dataset. Note that the scale of the values in (B) and (C) is dependent on the input image, and should not be assigned an absolute interpretation.

Importantly the GC-LDA model is able to produce probabilistic estimates of word and activation distributions for entirely new data points. Moreover, because each topic is associated with both a word distribution and a spatial distribution, we can proceed bidirectionally—either translating arbitrary text into image space, or decoding activations or images for their associated semantic content. Fig 7 illustrates three different applications of this approach. First, we can generate estimated activation probabilities for any word or set of words. Fig 7A illustrates three concrete examples. In (1), we observe a complex, distributed pattern of activity for the term 'motor', including activations in primary and supplementary motor cortices, cerebellum, and the basal ganglia. This result demonstrates that even though each topic in our dictionary is spatially constrained, individual words will often still have widely distributed neural correlates by virtue of loading on multiple topics.

In (2) we pass in a list of generic cognitive effort-related terms ('effort', 'difficult', and 'demands'), and observe highly circumscribed activations in frontoparietal regions frequently implicated in general goal-directed processing [32,53]. This result demonstrates the GC-LDA model’s ability to produce topics with relatively abstract semantics: while few studies explicitly set out to study the neural correlates of task difficulty or cognitive effort, our model successfully learns that regions like anterior insula and preSMA—which tend to activate in a very wide range of studies—likely support fairly general cognitive operations non-selectively invoked by many different tasks [Neurosynth; 9,cf. 14,54].

Lastly, in (3), we provide a full sentence as input ("painful stimulation during a language task"), producing a map with peaks in both pain-related (e.g., posterior insula) and language-related left perisylvian regions. While the model follows the bag-of-words assumption (i.e., the order of words has no effect on the generated image), its compositional character is evident, in that it is possible to generate a predicted image for virtually any cognitive state or states that can be described in text.

Second, we can generate a list of plausible semantic associates for any set of discrete brain coordinates. Fig 7B illustrates how this approach can be used to probe the function of a particular region both in isolation and in context. The top row lists the top probabilistic word associates for a temporoparietal region centered on MNI x-y-z coordinates (-56, -52, 18). The function of this region appears ambiguous—likely reflecting the presence of multiple overlapping neural circuits—with the top associates including 'reading', 'mentalizing', and 'pictures'. However, adding other coordinates strongly constrains functional interpretation. The addition of medial parietal (0, -58, 38) and dorsomedial prefrontal (4, 54, 26) activations (middle row) produces strong overall loadings on default network-related terms such as 'self', 'social', and 'moral'. By contrast, adding left superior temporal sulcus (-54, -40, 0) and left inferior frontal gyrus (-50, 26, 6) activations (bottom row) instead produces strong loadings on language and reading-related terms (Fig 7B). Thus, the GC-LDA model allows researchers to freely explore structure-function mappings in the brain in a context-specific way that recognizes that the cognitive operations supported by individual regions can contribute to multiple distinct cognitive functions.

Lastly, and perhaps most powerfully, the activation-to-word mapping approach can be generalized to entire whole-brain images. Given any real-valued input image, we can use the GC-LDA topics to generate a rank-ordered list of associated terms. While the output values cannot be interpreted as actual probabilities (due to the arbitrary scale of the inputs), the results are highly informative, providing a quantitative, literature-based decoding of virtually any pattern of whole-brain activity. Fig 7C illustrates the results for selected images, including two of the cognitive components from Yeo et al. [33], two of the BrainMap ICA components from Smith et al. [15], and two group-level HCP task contrasts (for additional results, see S2 Fig and S3 Fig). The decoded term list converges closely with extensive prior work; for example, a BrainMap ICA component focused largely on extrastriate visual cortex and adjacent inferotemporal areas is associated with motion, face perception, and other vision-related terms; a cognitive component from Yeo et al. [33] largely co-extensive with the frontoparietal control network loads most strongly on terms like “working memory”, “demands”, and “numerical”, and so on.

To more formally assess the performance of the decoder in an unbiased way, we used a set of NeuroVault images that were previously manually annotated using labels derived from the Cognitive Atlas ontology [55]. For each image, we used the image-to-text decoder to generate an image-specific rank-ordering of the 1,000 most common terms in the entire Neurosynth corpus. We then identified the rank, within that list, of each human-annotated Cognitive Atlas label. The median rank across all 300 images was 220—an impressive value considering the open-ended nature of the task and the unfiltered nature of the NeuroVault database (i.e., there is no guarantee that the images uploaded to Neurovault actually reflect the processes they are intended to reflect—a point we discuss further in the next section). By comparison, when we generated a null distribution of 1,000 permutations and computed the same median statistic, the mean and minimum values across all permutations were 442 and 384, respectively. In other words, the decoder produced rankings that were vastly more similar to expert human judgments than one would expect by chance.

### Brain decoding in context

**Fig 8:**
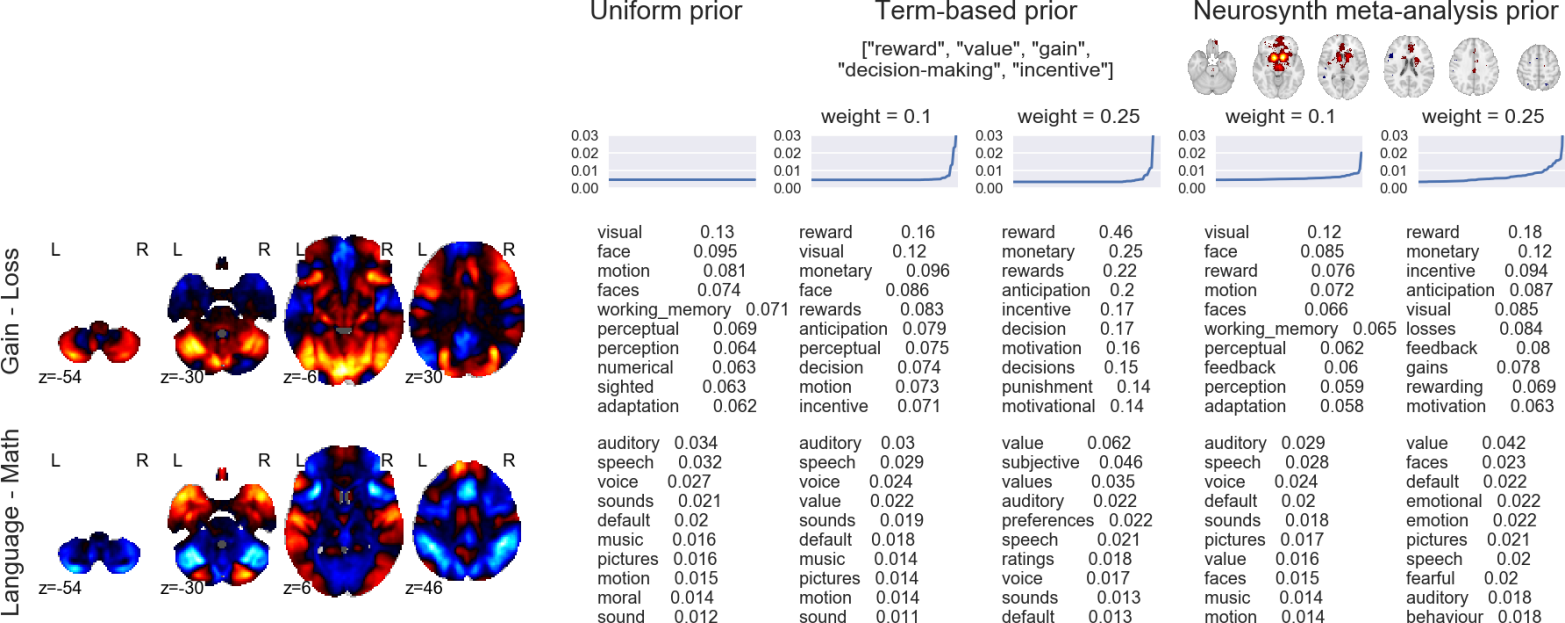
Effects of different topic priors on decoding results. The top 10 terms produced by the decoder are displayed for two different HCP contrasts (Gain > Loss from the Gambling task and Language > Math from the Language task) and three different sets of topic priors (left: uniform prior; middle: priors seeded with a list of reward-related terms; right: priors seeded with the Neurosynth “reward” meta-analysis map). For the non-uniform priors, results are displayed for priors of differing strengths (weak = 0.1, strong = 0.25). Line plots above the decoder outputs illustrate the prior distribution of topics used in each analysis (for the sake of visual clarity, topics are ordered by increasing weight separately in each case).

Importantly, the above analysis provides a necessarily conservative estimate of the performance of our decoder, because in many cases, the discrepancy between human-annotated and automatically-decoded labels is bound to reflect error in the former rather than the latter. We note that human-generated annotations typically reflect researchers' beliefs about which cognitive processes a particular experimental manipulation is *supposed* to influence, and do not represent ground truth. For example, the HCP Gambling Task [adapted from 56] was putatively designed "to assess reward processing and decision making" [57]. Yet the contrast between the reward and loss conditions (depicted in Fig 8) reveals robust reward-related increases in visual and frontoparietal cortices (Fig 8, top). Not surprisingly, terms like 'visual', and 'working memory' are at the top of the list returned by our decoder (see “uniform prior” results in Fig 8). Does this mean that the decoder is performing poorly, and failing to recover a known ground truth? No. Given the non-canonical pattern of observed brain activity, we believe a more plausible alternative is that the manipulation in question simply had a more complex effect on cognition than the "Reward vs. Loss" label might lead one to expect. In other words, the "assumption of pure insertion"—i.e., that the gain vs. loss contrast measures only cognitive processes related to reward or loss processing—is probably unwarranted in this case, as in many others [58,59].

The potential for discrepancy between expert human judgment and automated decoding creates an interesting conundrum: which answer should a neuroimaging researcher trust? Our view is that there is no blanket answer to this question; much depends on the particular context. Importantly, our decoding framework provides a way to quantitatively synthesize researchers’ prior beliefs with the associations learned by the GC-LDA topic model by explicitly manipulating the prior probabilities of the 200 topics. Because our model allows for bi-directional decoding (text-to-image or image-to-text), topic priors can be set by “seeding” the model with either a whole-brain image (or images), or a set of terms. The seeds are decoded in the normal way to update the initial uniform prior, and subsequent decoding is then based on the updated (non-uniform) priors. The approach is illustrated in Fig 8, which displays the results of a topic decoding analysis for two HCP task contrasts when the decoder is seeded (i) with uniform priors, (ii) with a set of reward-related terms, or (iii) with the whole-brain Neurosynth meta-analysis map for the term “reward” (http://neurosynth.org/analyses/terms/reward). The strength of the prior is also explicitly varied.

The major result illustrated in Fig 8 is that if one is able to specify a prior belief about the experimental context, the decoder respects this prior and produces results that are, to varying degrees, biased in the direction of the prior. The decoder results are implicitly smoothed by the underlying latent topics; for instance, in the top row of Fig 8, the terms “monetary” and “anticipation” appear near the top of the text-seeded results, even though they were not included in the list of seed terms. Moreover, the priors do not overwhelm the data (unless the strength parameter is set very high, as in the columns with weight = 0.25). When the reward-related priors are applied to a map that is highly inconsistent with the prior—as in the Language > Math contrast in the bottom row of Fig 8—the change in decoder results is much more subtle. Thus, our decoding framework provides a quantitative way of contextualizing interpretations of fMRI data in a principled way—or, alternatively, assessing the degree to which a particular interpretation is dependent on typically unstated prior beliefs.

## Discussion

The present work significantly advances beyond previous efforts with respect to both (a) the modeling of the latent structure of neurocognition and (b) the open-ended decoding of human brain activity. With respect to the former, the GC-LDA topic model we developed introduces several innovative features to the literature. First, the simultaneous use of spatial and semantic information allows the model to learn topics that have both well-localized spatial representations, and clear semantic correlates. Approximately half of the 200 topics we extracted in a completely data-driven way closely tracked previous functional and anatomical distinctions reported in previous fMRI studies. Second, the probabilistic nature of the resulting topics stands in contrast to many previous clustering and parcellation approaches, and more accurately reflects the many-to-many nature of the relationship between cognitive constructs and neurobiological structures. Third, the GC-LDA model's spatial symmetry constraint enabled us to generate brain-wide, data-driven estimates of the relative hemispheric lateralization of distinct cognitive topics. Consistent with the broader literature, most topics displayed a high degree of symmetry, with notable exceptions including the strong left-lateralization of language- and memory-related topics, and the more modest right-lateralization of response inhibition and face-related topics. Finally, the spatially compact, semantically well-defined nature of the 200 extracted topics makes the full topic set an ideal basis set for use in dimensionality reduction and image interpretation applications (as exemplified by the “topic reconstruction” analyses reported in the Supporting Results and illustrated in S4–S7 Figs).

From the standpoint of efforts to decode whole-brain activation patterns, our results also advances beyond previous work. First, by simultaneously constraining topics both spatially and semantically, the GC-LDA model generates topics designed to maximize the correspondence between cognition and brain activity. Conceptually, this idea is similar to other efforts that have sought to constrain factorizations of neuroimaging data using multiple sources of information (e.g., as in Yeo et al’s author-topic model; [33]). By contrast, most previous open-ended decoding unsupervised learning approaches have typically focused primarily or exclusively on a single level of analysis. That is, they have either focused on the factorization problem predominantly at the neurobiological level [36,39,60], and then (in some cases; e.g., [14,15,17]) projected the resulting components into the psychological/task space; or, they have done the converse, projecting predefined cognitive labels [e.g., 16] or semantically-derived components [e.g., 31] onto patterns of brain activity. Such approaches make sense in cases where researchers are deliberately privileging one level of analysis, but they are likely to produce suboptimal results when the goal is to derive the most parsimonious mappings *between* the cognitive and neurobiological levels of analysis.

Second, the generative nature of our decoding framework facilitates both encoding and decoding, enabling researchers not only to identify likely functional correlates of whole-brain activity patterns or sets of discrete activations, but also to project flexible text descriptions of tasks or processes into image space. This benefit is not unique to GC-LDA, of course; researchers have previously applied a variety of generative models to human brain imaging data [e.g., 61,62,63].

Third, our Bayesian approach allows researchers to formally specify priors on the GC-LDA topics, providing a powerful means of contextualizing interpretations and accounting for prior expectations and beliefs. We illustrate how a researcher can flexibly "seed" a decoding analysis using cognitive terms and/or whole-brain maps, thus ensuring that the decoder respects prior information about the experimental context. Current decoding approaches are typically forced to rely on unstated and inflexible assumptions about the base rates associated with different cognitive processes or tasks—a limitation that makes it difficult to know how much trust to place in a particular interpretation of one's results. While our approach currently has important limitations (see below), it represents an important step towards the goal of being able to decode arbitrary patterns of whole-brain activity in a way that formally synthesizes prior knowledge with observed results.

Naturally, the present work remains constrained by a number of important limitations. First, the specificity of the extracted topics is limited (both spatially and semantically) by the quality of the meta-analytic data in the Neurosynth database [for discussion, see 9]. In theory, greater specificity might be achievable using human-curated meta-analytic databases (e.g., BrainMap; [11]) or publicly deposited whole-brain images [64,65]. However, such resources are currently much smaller than Neurosynth—implying a significant decrement to the sensitivity of our data-intensive modeling approach—and, in the case of BrainMap, have usage restrictions that limit reproducibility and transparency. It is also important to recognize that—as illustrated by our results suggesting a discrepancy between expert human judgment and the accumulated literature—there is no guarantee that manually annotated data will be free of bias. Indeed, there should be little doubt that even an optimal coding of the primary literature would fail to remove a large source of bias, as the topics investigators study and the results they report are to a large degree inevitably influenced by their own beliefs as well as the historical trajectory of the discipline as a whole (e.g., the amygdala’s role in emotion may be overrepresented in the literature by virtue of researcher expectations, selection bias, etc.). Nevertheless, it is clear that the present topics already converge closely with prior literature. Moreover, the integration of our topics with the public NeuroVault repository ensures that researchers will always be able to apply the most current topic sets to their data at the push of a button.

Second, the output of the GC-LDA model is necessarily data-, context-, and assumption-dependent. While the topics produced by the model generally have parsimonious interpretations that accord well with previous findings, they should be treated as a useful, human-comprehensible approximation of the true nomological network of neurocognition, and not as a direct window into reality. For the sake of analytical tractability, our model assumes a one-to-one mapping between semantic representations and brain regions, whereas the underlying reality almost certainly involves enormously complex many-to-many mappings. Similarly, re-running the GC-LDA model on different input data, with different spatial priors, a different number of topics, or with different analysis parameters would necessarily produce somewhat different results. Of course, this concern applies equally to other large-scale data-driven approaches. We highlight it here simply because we would not want researchers to reify the topics we introduce here as if they are uniquely “real”. In our view, the overriding evaluation metric for any novel parcellation or clustering technique is whether it is scientifically productive over the long term [cf. 66]. With that caveat in mind, we believe that the framework introduced here strikes an excellent balance between interpretability, flexibility, and ease of use, and provides an important complement to previous data-driven approaches.

Third, our GC-LDA model, like most topic models, is completely unsupervised; it seeks to identify statistically parsimonious groupings and mappings of terms and activation patterns into cohesive topics, and makes no attempt to maximize classification or prediction accuracy on any supervised task. A natural consequence of this decision is that while topics extracted with GC-LDA are likely to be useful in a wide range of predictive applications (see Fig 7; Supporting Results; [35]), the model is very likely be outperformed in any given application by many other models that are specifically trained to optimize the criterion in question. We view this as a feature and not a bug, as our goal is to extract a set of parsimonious topics that simultaneously respect semantic and spatial constraints, and not to maximize predictive accuracy on any one task. However, it is important to recognize the inherent tradeoff implied by this choice, and in cases where predictive accuracy is paramount, we encourage researchers to use more traditional supervised approaches. A number of recent efforts have also sought to develop partly supervised approaches that couple an unsupervised dimensionality reduction step with a supervised learning step [e.g., 67,68,69] in the hopes of providing the best of both worlds (though the success of such an approach then depends on the quality and representativeness of the supervised tasks).

Lastly, while our decoding framework is based on probabilistic GC-LDA topics, the outputs it generates cannot typically be interpreted as probabilities, because the input images researchers conventionally seek to decode are mostly real-valued t or z maps whose meaning can vary dramatically. While this restriction limits the utility of our framework, it is, at present, unavoidable. Providing meaningful absolute estimates of the likelihood of different cognitive processes given observed brain activity would require either (a) that researchers converge on a common standard for representing observed results within a probabilistic framework (e.g., reporting the probability of subjects displaying supra-threshold activation in every voxel), or (b) re-training the GC-LDA model and associated decoding framework on a very large corpus of whole-brain images comparable to those that researchers seek to decode, rather than on a coordinate-based meta-analytic database. Of these two alternatives, we view the latter as the more feasible and productive strategy. We thus believe that the best hope for truly open-ended, fully probabilistic brain decoding lies in the widespread communal adoption of whole-brain images repositories like NeuroVault.org. We are optimistic that in the relatively near future, we will be able to use the topic modeling and decoding methods introduced here to produce highly informative, context-sensitive predictions about the mental processes implied by arbitrary patterns of whole-brain activity.

## Materials and methods

### Datasets

All data used to train the GC-LDA topic model came from the Neurosynth database [9; neurosynth.org]. The database contains activation coordinates that were automatically extracted from 11,409 published fMRI studies (release v0.6, July 2015; data are available from github.com/neurosynth/neurosynth-data), as well as associated semantic terms extracted from the corresponding article abstracts. Further details have been reported in previous studies [9,14,31,70].

For decoding analyses, we used whole-brain maps obtained from several sources, including: (1) *The 500-subject release of the Human Connectome Project* *[71]*. We focused on single-subject whole-brain beta maps from several functional tasks. In all cases, we used experimental contrasts predefined by the HCP research team and included in the “preprocessed” data release (i.e., we did not preprocess or alter the provided contrast images in any way, nor did we filter participants for relatedness or any other criterion). Studied contrasts included the comparison between faces and shapes in the Emotion task; between language and math conditions in the Language-Math condition; between social and non-social motion in the Social Cognition task. (2) *NeuroVault*.*org maps*. We downloaded two sets of maps from the NeuroVault whole-brain image repository: (i) a completely random set of 100 images (subject to the constraint that each image had to come from a different image collection, to maximize independence of images), and (ii) a random set of 300 NeuroVault images that had been previously manually annotated using the Cognitive Atlas ontology for a completely different purpose (Sochat et al., in preparation). (3) *BrainMap ICA and Yeo et al*. *author-topic “cognitive component” maps*. We obtained these two sets of maps—reported in Smith et al. [15] and Yeo et al. [33], respectively—via the web.

All analyses were conducted in the standard MNI152 2mm space. Images that were not nominally in this space (e.g., many of the NeuroVault maps) were transformed to the target using an affine transformation with continuous interpolation (using the *resample_img* function in the nilearn package; nilearn.github.io). Such transformations are imperfect and subject to considerable error, but we nevertheless opted for the simplicity of such an approach seeing as our goal was to illustrate the application of the GC-LDA model rather than to draw concrete inferences about any of the tested images. Once in the MNI152 space, we used the standard gray matter mask from the FSL package [72] to select voxels for analysis.

### Topic modeling

**Fig 9:**
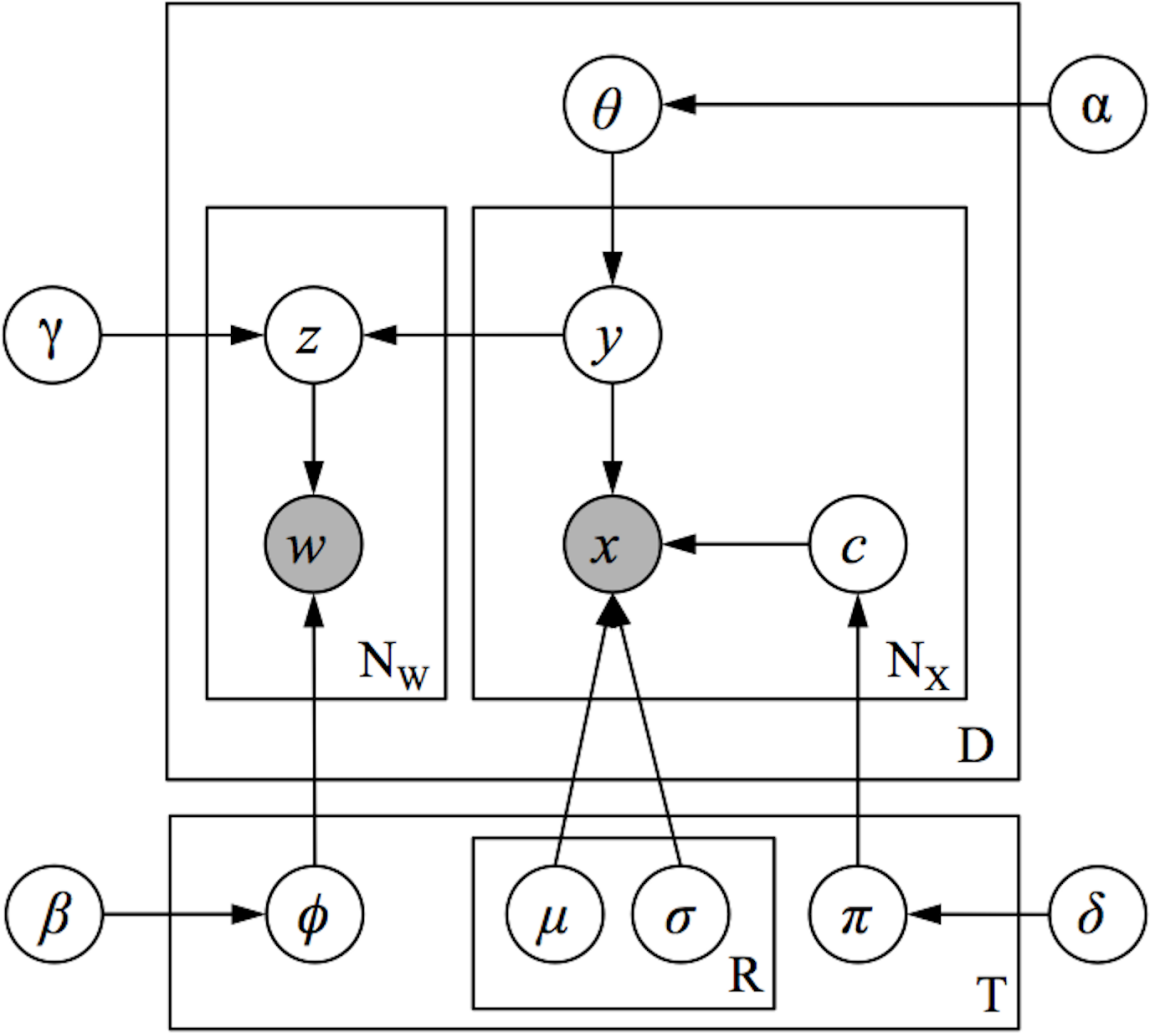
Graphical model of the full GC-LDA model.

A high-level schematic of the model we employ is presented in Fig 2; the model is presented using graphical model plate notation representation in Fig 9. We begin with the Neurosynth dataset, which contains data extracted from 11,406 published fMRI articles. Each of the 11,406 document consists of (1) a set of unigrams and bigrams of words extracted from the publication's abstract, describing what each experiment was about, and (2) the set of peak-activation coordinates that were reported in HTML tables within the paper (for data extraction details, see Yarkoni et al, 2011). The model learns a set of *T topics*, where each topic is associated with some spatial distribution (e.g., a 3-dimensional Gaussian distribution with parameters *μ*_*t*_ and *σ*_*t*_), and a multinomial distribution *ϕ*_*t*_ over all of the unique types of linguistic features (consisting of unigrams and bigrams) in the corpus. This model is a generative model, meaning that it learns the joint probability distribution of all variables, and thus describes a process that can produce new approximations of the observed data (the linguistic features and activation coordinates) via a set of latent (unobserved) topics.

The model assumes that each document *d* is generated by first sampling a multinomial probability distribution *θ*_*d*_ over topics from a Dirichlet prior distribution. Then, to generate each activation peak *x* in the document, the document first samples a topic *y* from its distribution over topics *θ*_*d*_ and then samples a peak activation at location *x* from the spatial distribution associated with topic *y*. To generate each word in the document, a topic *z* is sampled proportional to the number of times that the document sampled activations peaks from each topic, and then a word token *w* is sampled from topic *z*’s probability distribution over word types *ϕ*_*z*_. To illustrate this process, consider the example Document 1 shown in Fig 2, which we can imagine describes an experiment measuring reaction times on a word-identification task. The model assumes that neural activation peaks reported in this experiment will be sequentially sampled from the spatial distributions associated with topics 1 and 2 (which relate to language processes and motor processes, respectively). The model then assumes that the words in the document—used to describe the experiment and its results—will be sampled from the linguistic distributions associated with topics 1 and 2, proportional to the number of times activation peaks were sampled from each of these topics.

Because the model enforces a correspondence between the frequency with which documents sample their words and activations from each topic, the model ensures that over the document corpus, the linguistic features associated with each topic will be closely related to the topic’s spatial distribution over activations. More specifically, the model will identify a topic-specific distribution over neural activations that tends to co-occur with the topic’s linguistic features across the corpus.

The general framework of the GC-LDA model allows the experimenter to choose any valid probability distribution for the spatial component of each topic. The results displayed in Figs 3–8 correspond to a GC-LDA model in which each topic's spatial distribution is captured by a mixture of two Gaussian distributions that have been constrained to be symmetric about the x-axis. In our experiments, we evaluated several variations of the GC-LDA model using different probability distributions. We started with each topic having a single multidimensional Gaussian spatial distribution. We then replaced the single Gaussian distribution with a Gaussian Mixture distribution containing two components (i.e. subregions). In a further variant of this model (pictured in Fig 1), we constrained the spatial arrangement of the two component distributions of the Gaussian mixture distribution, such that their means were symmetrical with respect to the x-axis of the brain (i.e., so that for each topic, the spatial distribution would consist of one component region in the left hemisphere and a second component region in the right hemisphere). This allowed us to include an anatomical constraint based on known features of functional neuroanatomy—specifically the fact that there is generally a bilateral symmetry with respect to neural functionality. It further provided us with an automated way of measuring the lateral asymmetry of different cognitive functions (given by each topic's probability of drawing an activation from its different components).

Given a formalized generative process for any of these models, we can use Bayesian inference methods to learn all of the latent (unobserved) parameters of this model from the observed data (see [35], for details). Specifically, the model learns a set of *T* topics, where each topic has an associated spatial probability distribution over the coordinates in the brain, as well as a multinomial distribution over linguistic features. The model additionally learns the topic mixture weights for each document.

Although Bayesian statistics can have poor scaling behavior with respect to the number of observed variables *p* [73], inference for GC-LDA is well behaved with respect to *p*. For the GC-LDA inference methods described in [33], the computational complexity per iteration is *O*(*T*(*N*_*W*_ + *N*_*X*_*R*)), where *T* is the number of topics, *R* is the number of subregions, and *N*_*W*_ and *N*_*X*_ are the number of word tokens and peak activation tokens, respectively.

### Text-to-image and image-to-text decoding

For text-to-image decoding (Fig 7A), we first compute a Topic (*T*) × Word-type (*W*) matrix of conditional probabilities *P*_*T*×*W*_, where cell *P*_*ij*_ is the probability *p*(*t* = *i*|*w* = *j*) that the model assigns word type *w* from the text input to the *i*^th^ topic. This matrix is computed, using Bayes’ rule, from the topic’s probability distributions over word types *Φ*_*W*×*T*_ in the trained GC-LDA model, as follows:

$$P_{tw}=p(t=i|w=j)=\frac{p(w=j|t=i)\cdot p(t=i)}{p(w)}=\frac{\Phi_{ji}}{\sum_{i=1}^{T}\Phi_{ji}}$$

assuming a uniform prior probability of each topic, *p*(*t*). We then obtain a vector of topic weights ***τ*** for the entire input by summing over the all word tokens *w* in the input; i.e.,

$$\tau_{t}=\sum_{w}P_{tw}$$

Lastly, we multiply this vector of topic weights by a Topic (T) × Voxel (V) matrix *A*_*T*×*V*_, where cell *A*_*ij*_ reflects the smoothed conditional probability *p*(*v* = *j*|*t* = *i*) that the model samples an activation at brain voxel *j* (of *V* total voxels) from topic *i*. The rows of this matrix are (smoothed versions of) the images displayed in Fig 3. The resulting (vectorized) whole-brain image is thus given by the product: ***τ*** ∙ *A*.

Note that the resulting values cannot be interpreted as probabilities, because we deliberately sum over words in the input rather than computing the joint probability. The reason for this is that, while the latter approach is technically feasible, it typically produces very similar results for short inputs, and produces unstable results when the input sentence exceeds a few words in length (because the sparse nature of the word-to-topic mapping results in the compounding of many very small probabilities).

For discrete coordinate-to-text decoding (Fig 7B), we repeat the above process, but proceed in the opposite direction. That is, we first compute a Topic × Voxel matrix *P*_*T*×*V*_, where cell *P*_*ij*_ reflects the conditional probability *p*(*t* = *i*|*v* = *j*) that the model assigns the activation at voxel *j* in the input to the *i*^th^ topic. This matrix is computed from the trained GC-LDA model by first computing the empirically observed probability of sampling of each voxel from each topic (given each topic’s spatial distribution), and then renormalizing these probabilities using Bayes’ rule, under the same uniform prior assumption used above for text-to-image decoding (i.e., we compute the conditional probability *p*(*t* = *i*|*v* = *j*) as if the prior, *p*(*t*), was uniform over all topics). We then sum over all of the input activations to obtain a vector of topic weights ***τ*** for the given input. Lastly, we project the topic weights into the word space by multiplying the vector of topic weights ***τ*** by the Topic × Word matrix *Φ*_*W*×*T*_, where cell *Φ*_*ij*_ reflects the conditional probability *p*(*w* = *i*|*t* = *j*) that the model samples the *i*^*th*^ word type from topic *j*: ***τ*** ∙ *Φ*.

To decode text from continuous whole-brain images (Fig 7C), a slightly different approach is required. Although whole-brain decoding superficially resembles the decoding of discrete coordinates, the fact that the input images are real-valued and have arbitrary scaling precludes a true probabilistic treatment. Instead, we adopt a modified approach that weights the conditional probability matrix *P*_*T*×*V*_ by the similarity of the input image to each of the GC-LDA topic maps. We compute a vector of topic weights ***τ*** as:

$$\tau_{t}=P_{T\times V}\cdot I_{V}$$

where *P*_*T*×*V*_ is the Topic × Voxel matrix of conditional probabilities of assigning an activation at voxel *v* to topic *t*, and *I*_*V*_ is the vectorized whole-brain input image. We then project the topic weights into word space in the same way as for the discrete coordinates: ***τ*** ∙ *Φ*. The scale of the resulting values is arbitrary, and depends on the input image, but the rank-ordering of terms is instructive and typically converges closely with human-annotated labels.

### Contextual decoding via topic “seeding”

Specifying priors on the GC-LDA topics can in principle be accomplished directly, by simply setting the desired prior probabilities on topics *p*(*t*) when computing the matrices *P*_*T*×*V*_ and *P*_*T*×*W*_. The decoder results will then directly reflect the adjustment in both the text-to-image and image-to-text directions. However, researchers are unlikely to have strong intuitions about the relative base rates of the latent topics themselves. More commonly, they will instead wish to update the priors indirectly, based on a more intuitive expression of the experimental context or prior belief. This can be accomplished by “seeding” the priors with image and/or text inputs. In this case, the procedure can be thought of as a two-step application of the decoding methods described above. On the first pass, the input image or text is used to estimate values of ***τ*** (no further output is generated). On the second pass, the ***τ*** computed during the first pass is used as an informative prior *p*(*t*) in computing matrix *P*_*T*×*V*_ or *P*_*T*×*W*_ as described previously, and this updated matrix is applied to the actual image or text to be decoded. This procedure can repeat an indefinite number of times, as in a typical Bayesian context (i.e., the posterior ***τ*** probabilities become the priors for the next decoding application).

### Software

All of the methods and analyses were implemented in the Python programming language. We used the standard scientific Python stack for analysis: Numpy [74] as the basis for all numerical computing routines, Scipy [75] for various scientific utilities, and pandas for structured data manipulation [76]. For neuroimaging data analysis, we used the nibabel library for I/O operations and basic image manipulation (http://nipy.org/nibabel) and the nilearn library (http://nilearn.github.io) to plot brain slices. Word clouds were generated using the word_cloud package (https://github.com/amueller/word_cloud). An open-source implementation of our GC-LDA model, including documentation and examples, is publicly available on GitHub (https://github.com/timothyrubin/python_gclda). The topic maps reported here are available as a collection of interactive, downloadable whole-brain maps from the NeuroVault website (http://neurovault.org).

## Supporting information

S1 Supporting Results Whole-brain image reconstruction using learned GC-LDA topics. (PDF)

S1 Fig Full results for all topics learned by the GC-LDA model. Each row represents a single topic. For each topic, the word cloud displays the top semantic associates (the size of each term is roughly proportional to the strength of its loading, and the orthviews display all hard assignments of activations to that topic (each point represents a single activation from a single study in Neurosynth).
(JPG)

S2 Fig Topic-based decoding of 20 BrainMap-derived ICA components reported in Smith et al. [15]. (JPG)

S3 Fig Topic-based decoding of 12 “cognitive components” reported in Yeo et al. [33]. (JPG)

S4 Fig Topic-based reconstruction of whole-brain activity maps. Representative examples from (A) the set of 20 BrainMap ICA components reported in Smith et al. [15]. (B) the NeuroVault whole-brain image repository [2], and (C) single-subject contrast maps from the emotion processing task in the Human Connectome Project dataset (face vs. shape contrast). Each row displays the original (left) and reconstructed (center) image, along with the coefficient of determination (R^2^) for the fitted reconstruction model, and a scatter plot of all voxels (right).
(JPG)

S5 Fig Reconstruction of 20 BrainMap ICA components reported in Smith et al. [15]. (JPG)

S6 Fig Topic reconstruction of 12 “cognitive components” reported in Yeo et al. [33]. (JPG)

S7 Fig Topic reconstruction of 100 random maps extracted from the NeuroVault whole-brain image repository. Labels in white indicate human-annotated cognitive atlas paradigm, when available.
(JPG)

## References

[1] Haynes J-D, Rees G. Decoding mental states from brain activity in humans. Nat Rev Neurosci. 2006;7: 523–534. doi: 10.1038/nrn1931 16791142 10.1038/nrn1931

[2] Kriegeskorte N, Kievit RA. Representational geometry: integrating cognition, computation, and the brain. Trends Cogn Sci. 2013;17: 401–412. doi: 10.1016/j.tics.2013.06.007 23876494 10.1016/j.tics.2013.06.007 PMC3730178

[3] Haxby JV, Connolly AC, Guntupalli JS. Decoding neural representational spaces using multivariate pattern analysis. Annu Rev Neurosci. 2014;37: 435–456. doi: 10.1146/annurev-neuro-062012-170325 25002277 10.1146/annurev-neuro-062012-170325

[4] Mitchell TM, Hutchinson R, Niculescu RS, Pereira F, Wang X, Just M, et al Learning to decode cognitive states from brain images Mach Learn. Springer; 2004;57: 145–175.

[5] Mitchell TM, Shinkareva SV, Carlson A, Chang KM, Malave VL, Mason RA, et al Predicting human brain activity associated with the meanings of nouns. Science. AAAS; 2008;320: 1191. 10.1126/science.1152876 18511683

[6] Cox DD, Savoy RL. Functional magnetic resonance imaging (fMRI)’'brain reading'': detecting and classifying distributed patterns of fMRI activity in human visual cortex. Neuroimage. Elsevier; 2003;19: 261–270. 10.1016/s1053-8119(03)00049-1 12814577

[7] Poldrack RA, Halchenko YO, Hanson SJ. Decoding the large-scale structure of brain function by classifying mental states across individuals. Psychol Sci. SAGE Publications; 2009;20: 1364–1372. 10.1111/j.1467-9280.2009.02460.x PMC2935493 19883493

[8] Shirer WR, Ryali S, Rykhlevskaia E, Menon V, Greicius MD. Decoding subject-driven cognitive states with whole-brain connectivity patterns. Cereb Cortex. 2012;22: 158–165. doi: 10.1093/cercor/bhr099 21616982 10.1093/cercor/bhr099 PMC3236795

[9] Yarkoni T, Poldrack RA, Nichols TE, Essen V, David C, Wager TD. Large-scale automated synthesis of human functional neuroimaging data Nat Methods. Nature Publishing Group; 2011;8: 665–670. doi: 10.1038/nmeth.1635 21706013 10.1038/nmeth.1635 PMC3146590

[10] Laird AR, Eickhoff SB, Fox PM, Uecker AM, Ray KL, Saenz JJ, et al The BrainMap strategy for standardization, sharing, and meta-analysis of neuroimaging data BMC Res Notes. BioMed Central Ltd; 2011;4: 349 doi: 10.1186/1756-0500-4-349 21906305 10.1186/1756-0500-4-349 PMC3180707

[11] Laird AR, Lancaster JL, Fox PT. BrainMap: the social evolution of a human brain mapping database. Neuroinformatics. 2005;3: 65–78. 15897617 10.1385/ni:3:1:065

[12] Poldrack RA. Can cognitive processes be inferred from neuroimaging data. Trends Cogn Sci. 2006;10: 59–63. doi: 10.1016/j.tics.2005.12.004 16406760 10.1016/j.tics.2005.12.004

[13] Poldrack RA. Inferring Mental States from Neuroimaging Data: From Reverse Inference to Large-Scale Decoding Neuron. Elsevier Inc.; 2011;72: 692–697. doi: 10.1016/j.neuron.2011.11.001 22153367 10.1016/j.neuron.2011.11.001 PMC3240863

[14] Chang LJ, Yarkoni T, Khaw MW, Sanfey AG. Decoding the Role of the Insula in Human Cognition: Functional Parcellation and Large-Scale Reverse Inference. Cereb Cortex. 2012; doi: 10.1093/cercor/bhs065 22437053 10.1093/cercor/bhs065 PMC3563343

[15] Smith SM, Fox PT, Miller KL, Glahn DC, Fox PM, Mackay CE, et al Correspondence of the brain’s functional architecture during activation and rest Proc Natl Acad Sci U S A. National Academy of Sciences; 2009;106: 13040–13045. doi: 10.1073/pnas.0905267106 19620724 10.1073/pnas.0905267106 PMC2722273

[16] Andrews-Hanna JR, Saxe R, Yarkoni T. Contributions of episodic retrieval and mentalizing to autobiographical thought: evidence from functional neuroimaging, resting-state connectivity, and fMRI meta-analyses. Neuroimage. 2014;91: 324–335. doi: 10.1016/j.neuroimage.2014.01.032 24486981 10.1016/j.neuroimage.2014.01.032 PMC4001766

[17] Bzdok D, Langner R, Schilbach L, Jakobs O, Roski C, Caspers S, et al Characterization of the temporo-parietal junction by combining data-driven parcellation, complementary connectivity analyses, and functional decoding. Neuroimage. 2013;81: 381–392. doi: 10.1016/j.neuroimage.2013.05.046 23689016 10.1016/j.neuroimage.2013.05.046 PMC4791053

[18] Naselaris T, Kay KN, Nishimoto S, Gallant JL. Encoding and decoding in fMRI. Neuroimage. 2011;56: 400–410. doi: 10.1016/j.neuroimage.2010.07.073 20691790 10.1016/j.neuroimage.2010.07.073 PMC3037423

[19] Vigneau M, Beaucousin V, Herve PY, Duffau H, Crivello F, Houde O, et al Meta-analyzing left hemisphere language areas: Phonology, semantics, and sentence processing. Neuroimage. 2006; 10.1016/j.neuroimage.2005.11.002 16413796

[20] Buhle JT, Silvers JA, Wager TD, Lopez R, Onyemekwu C, Kober H, et al Cognitive reappraisal of emotion: a meta-analysis of human neuroimaging studies. Cereb Cortex. 2014;24: 2981–2990. doi: 10.1093/cercor/bht154 23765157 10.1093/cercor/bht154 PMC4193464

[21] Swick D, Ashley V, Turken U. Are the neural correlates of stopping and not going identical? Quantitative meta-analysis of two response inhibition tasks. Neuroimage. 2011;56: 1655–1665. doi: 10.1016/j.neuroimage.2011.02.070 21376819 10.1016/j.neuroimage.2011.02.070

[22] Beckmann M, Johansen-Berg H, Rushworth MFS. Connectivity-based parcellation of human cingulate cortex and its relation to functional specialization. J Neurosci. 2009;29: 1175–1190. doi: 10.1523/JNEUROSCI.3328-08.2009 19176826 10.1523/JNEUROSCI.3328-08.2009 PMC6665147

[23] Blei DM, Ng AY, Jordan MI. Latent Dirichlet Allocation. J Mach Learn Res. 2003;3: 993–1022.

[24] Blei DM. Probabilistic topic models. Commun ACM. 2012;55: 77.

[25] Mcauliffe JD, Blei DM. Supervised Topic Models In: Platt JC, Koller D, Singer Y, Roweis ST, editors. Advances in Neural Information Processing Systems 20. Curran Associates, Inc.; 2008 pp. 121–128.

[26] Rubin TN, Chambers A, Smyth P, Steyvers M. Statistical Topic Models for Multi-Label Document Classification. Corpus. 2011; 1–44.

[27] Zhai C, Chengxiang Z, John L. Model-based feedback in the language modeling approach to information retrieval. Proceedings of the tenth international conference on Information and knowledge management—CIKM’01. 2001. doi:10.1145/502585.502654

[28] Cao L, Liangliang C, Li F-F. Spatially Coherent Latent Topic Model for Concurrent Segmentation and Classification of Objects and Scenes. 2007 IEEE 11th International Conference on Computer Vision. 2007. doi:10.1109/iccv.2007.4408965

[29] Griffiths TL, Steyvers M. Finding scientific topics. Proceedings of the National Academy of Sciences. 2004;101: 5228–5235. 10.1073/pnas.0307752101 PMC387300 14872004

[30] Steyvers M, Smyth P, Rosen-Zvi M, Griffiths T. Probabilistic Author-topic Models for Information Discovery. Proceedings of the Tenth ACM SIGKDD International Conference on Knowledge Discovery and Data Mining. New York, NY, USA: ACM; 2004. pp. 306–315.

[31] Poldrack RA, Mumford JA, Schonberg T, Kalar D, Barman B, Yarkoni T. Discovering relations between mind, brain, and mental disorders using topic mapping. PLoS Comput Biol. 2012;8: e1002707 doi: 10.1371/journal.pcbi.1002707 23071428 10.1371/journal.pcbi.1002707 PMC3469446

[32] Duncan J. The multiple-demand (MD) system of the primate brain: mental programs for intelligent behaviour. Trends Cogn Sci. Elsevier; 2010;14: 172–179. doi: 10.1016/j.tics.2010.01.004 20171926 10.1016/j.tics.2010.01.004

[33] Yeo BTT, Thomas Yeo BT, Krienen FM, Eickhoff SB, Yaakub SN, Fox PT, et al Functional Specialization and Flexibility in Human Association Cortex. Cereb Cortex. 2014;25: 3654–3672. doi: 10.1093/cercor/bhu217 25249407 10.1093/cercor/bhu217 PMC4598819

[34] Blei DM, Jordan MI. Modeling annotated data. Proceedings of the 26th annual international ACM SIGIR conference on Research and development in informaion retrieval—SIGIR ‘03. 2003. doi:10.1145/860435.860460

[35] Rubin, T. N., Koyejo, O., Jones, M. N., & arkoni, T. Generalized Correspondence-LDA Models (GC-LDA) for Identifying Functional Regions in the Brain.

[36] Craddock RC, James GA, Holtzheimer PE 3rd, Hu XP, Mayberg HS. A whole brain fMRI atlas generated via spatially constrained spectral clustering. Hum Brain Mapp. 2012;33: 1914–1928. doi: 10.1002/hbm.21333 21769991 10.1002/hbm.21333 PMC3838923

[37] Blumensath T, Jbabdi S, Glasser MF, Van Essen DC, Ugurbil K, Behrens TEJ, et al Spatially constrained hierarchical parcellation of the brain with resting-state fMRI. Neuroimage. 2013;76: 313–324. doi: 10.1016/j.neuroimage.2013.03.024 23523803 10.1016/j.neuroimage.2013.03.024 PMC3758955

[38] Bellec P, Rosa-Neto P, Lyttelton OC, Benali H, Evans AC. Multi-level bootstrap analysis of stable clusters in resting-state fMRI. Neuroimage. 2010;51: 1126–1139. doi: 10.1016/j.neuroimage.2010.02.082 20226257 10.1016/j.neuroimage.2010.02.082

[39] Yeo BTT, Krienen FM, Sepulcre J, Sabuncu MR, Lashkari D, Hollinshead M, et al The organization of the human cerebral cortex estimated by intrinsic functional connectivity. J Neurophysiol. 2011;106: 1125–1165. doi: 10.1152/jn.00338.2011 21653723 10.1152/jn.00338.2011 PMC3174820

[40] Power JD, Cohen AL, Nelson SM, Wig GS, Barnes KA, Church JA, et al Functional network organization of the human brain. Neuron. 2011;72: 665–678. doi: 10.1016/j.neuron.2011.09.006 22099467 10.1016/j.neuron.2011.09.006 PMC3222858

[41] Mountcastle VB, Lynch JC, Georgopoulos A, Sakata H, Acuna C. Posterior parietal association cortex of the monkey: command functions for operations within extrapersonal space. J Neurophysiol. 1975;38: 871–908. 808592 10.1152/jn.1975.38.4.871

[42] Bushnell MC, Goldberg ME, Robinson DL. Behavioral enhancement of visual responses in monkey cerebral cortex. I. Modulation in posterior parietal cortex related to selective visual attention. J Neurophysiol. 1981;46: 755–772. 7288463 10.1152/jn.1981.46.4.755

[43] Snyder LH, Batista AP, Andersen RA. Coding of intention in the posterior parietal cortex. Nature. 1997;386: 167–170. doi: 10.1038/386167a0 9062187 10.1038/386167a0

[44] Andersen RA, Essick GK, Siegel RM. Encoding of spatial location by posterior parietal neurons. Science. 1985;230: 456–458. 4048942 10.1126/science.4048942

[45] Zald DH. The human amygdala and the emotional evaluation of sensory stimuli. Brain Res Brain Res Rev. 2003;41: 88–123. 12505650 10.1016/s0165-0173(02)00248-5

[46] LeDoux J. The emotional brain, fear, and the amygdala. Cell Mol Neurobiol. 2003;23: 727–738. 14514027 10.1023/A:1025048802629 PMC11530156

[47] Phelps EA. Emotion and cognition: insights from studies of the human amygdala. Annu Rev Psychol. 2006;57: 27–53. doi: 10.1146/annurev.psych.56.091103.070234 16318588 10.1146/annurev.psych.56.091103.070234

[48] Lindquist KA, Wager TD, Kober H, Bliss-Moreau E, Barrett LF. The brain basis of emotion: a meta-analytic review. Behav Brain Sci. 2012;35: 121–143. doi: 10.1017/S0140525X11000446 22617651 10.1017/S0140525X11000446 PMC4329228

[49] Kohn N, Eickhoff SB, Scheller M, Laird AR, Fox PT, Habel U. Neural network of cognitive emotion regulation—an ALE meta-analysis and MACM analysis. Neuroimage. 2014;87: 345–355. doi: 10.1016/j.neuroimage.2013.11.001 24220041 10.1016/j.neuroimage.2013.11.001 PMC4801480

[50] Dehaene S, Stanislas D, Laurent C. The unique role of the visual word form area in reading. Trends Cogn Sci. 2011;15: 254–262. doi: 10.1016/j.tics.2011.04.003 21592844 10.1016/j.tics.2011.04.003

[51] Kanwisher N, McDermott J, Chun MM. The fusiform face area: a module in human extrastriate cortex specialized for face perception. Journal of Neuroscience. 1997;17: 4302–4311. 9151747 10.1523/JNEUROSCI.17-11-04302.1997 PMC6573547

[52] Aron AR, Robbins TW, Poldrack RA. Inhibition and the right inferior frontal cortex. Trends Cogn Sci. 2004;8: 170–177. doi: 10.1016/j.tics.2004.02.010 15050513 10.1016/j.tics.2004.02.010

[53] Dosenbach NU, Visscher KM, Palmer ED, Miezin FM, Wenger KK, Kang HC, et al A core system for the implementation of task sets. Neuron. 2006;50: 799–812. doi: 10.1016/j.neuron.2006.04.031 16731517 10.1016/j.neuron.2006.04.031 PMC3621133

[54] Nelson SM, Dosenbach NUF, Cohen AL, Wheeler ME, Schlaggar BL, Petersen SE. Role of the anterior insula in task-level control and focal attention. Brain Struct Funct. Springer; 2010; 1–12. 10.1007/s00429-010-0260-2 PMC2886908 20512372

[55] Poldrack RA, Kittur A, Kalar D, Miller E, Seppa C, Gil Y, et al The Cognitive Atlas: Toward a Knowledge Foundation for Cognitive Neuroscience. Front Neuroinform. Frontiers Research Foundation; 2011;5: 11 doi: 10.3389/fninf.2011.00011 21922006 10.3389/fninf.2011.00017 PMC3167196

[56] Delgado MR, Nystrom LE, Fissell C, Noll DC, Fiez JA. Tracking the hemodynamic responses to reward and punishment in the striatum. J Neurophysiol. 2000;84: 3072–3077. 11110834 10.1152/jn.2000.84.6.3072

[57] Barch DM, Burgess GC, Harms MP, Petersen SE, Schlaggar BL, Corbetta M, et al Function in the human connectome: task-fMRI and individual differences in behavior. Neuroimage. 2013;80: 169–189. doi: 10.1016/j.neuroimage.2013.05.033 23684877 10.1016/j.neuroimage.2013.05.033 PMC4011498

[58] Friston KJ, Price CJ, Fletcher P, Moore C, Frackowiak RS, Dolan RJ. The trouble with cognitive subtraction. Neuroimage. 1996;4: 97–104. doi: 10.1006/nimg.1996.0033 9345501 10.1006/nimg.1996.0033

[59] Poldrack RA. Subtraction and Beyond: The Logic of Experimental Designs for Neuroimaging In: Hanson SJ, Bunzl M, editors. Foundational Issues in Human Brain Mapping. The MIT Press; 2010 pp. 147–160.

[60] Manning JR, Ranganath R, Norman KA, Blei DM. Topographic factor analysis: a Bayesian model for inferring brain networks from neural data. PLoS One. 2014;9: e94914 doi: 10.1371/journal.pone.0094914 24804795 10.1371/journal.pone.0094914 PMC4012983

[61] Brodersen KH, Schofield TM, Leff AP, Ong CS, Lomakina EI, Buhmann JM, et al Generative embedding for model-based classification of fMRI data. PLoS Comput Biol. 2011;7: e1002079 doi: 10.1371/journal.pcbi.1002079 21731479 10.1371/journal.pcbi.1002079 PMC3121683

[62] Huth AG, de Heer WA, Griffiths TL, Theunissen FE, Gallant JL. Natural speech reveals the semantic maps that tile human cerebral cortex. Nature. 2016;532: 453–458. doi: 10.1038/nature17637 27121839 10.1038/nature17637 PMC4852309

[63] van Gerven MAJ, de Lange FP, Heskes T. Neural decoding with hierarchical generative models. Neural Comput. 2010;22: 3127–3142. doi: 10.1162/NECO_a_00047 20858128 10.1162/NECO_a_00047

[64] Salimi-Khorshidi G, Smith SM, Keltner JR, Wager TD, Nichols TE. Meta-analysis of neuroimaging data: A comparison of image-based and coordinate-based pooling of studies. Neuroimage. Elsevier; 2009;45: 810–823. doi: 10.1016/j.neuroimage.2008.12.039 19166944 10.1016/j.neuroimage.2008.12.039

[65] Gorgolewski KJ, Varoquaux G, Rivera G, Schwarz Y, Ghosh SS, Maumet C, et al NeuroVault.org: a web-based repository for collecting and sharing unthresholded statistical maps of the human brain. Front Neuroinform. 2015;9: 8 doi: 10.3389/fninf.2015.00008 25914639 10.3389/fninf.2015.00008 PMC4392315

[66] Poldrack RA, Yarkoni T. From Brain Maps to Cognitive Ontologies: Informatics and the Search for Mental Structure. Annu Rev Psychol. 2015; doi: 10.1146/annurev-psych-122414-033729 26393866 10.1146/annurev-psych-122414-033729 PMC4701616

[67] Bzdok D, Eickenberg M, Grisel O, Thirion B, Varoquaux G. Semi-Supervised Factored Logistic Regression for High-Dimensional Neuroimaging Data In: Cortes C, Lawrence ND, Lee DD, Sugiyama M, Garnett R, editors. Advances in Neural Information Processing Systems 28. Curran Associates, Inc.; 2015 pp. 3348–3356.

[68] Bzdok D, Varoquaux G, Grisel O, Eickenberg M, Poupon C, Thirion B. Formal Models of the Network Co-occurrence Underlying Mental Operations. PLoS Comput Biol. 2016;12: e1004994 doi: 10.1371/journal.pcbi.1004994 27310288 10.1371/journal.pcbi.1004994 PMC4911040

[69] Michel V, Gramfort A, Varoquaux G, Eger E, Keribin C, Thirion B. A supervised clustering approach for fMRI-based inference of brain states. Pattern Recognit. 2012/6;45: 2041–2049.

[70] Pauli WM, O’Reilly RC, Yarkoni T, Wager TD. Regional specialization within the human striatum for diverse psychological functions. Proc Natl Acad Sci U S A. 2016;113: 1907–1912. doi: 10.1073/pnas.1507610113 26831091 10.1073/pnas.1507610113 PMC4763751

[71] Van Essen DC, Smith SM, Barch DM, Behrens TEJ, Yacoub E, Ugurbil K, et al The WU-Minn Human Connectome Project: an overview. Neuroimage. 2013;80: 62–79. doi: 10.1016/j.neuroimage.2013.05.041 23684880 10.1016/j.neuroimage.2013.05.041 PMC3724347

[72] Smith SM, Jenkinson M, Woolrich MW, Beckmann CF, Behrens TEJ, Johansen-Berg H, et al Advances in functional and structural MR image analysis and implementation as FSL. Neuroimage. Elsevier; 2004;23: S208–S219. doi: 10.1016/j.neuroimage.2004.07.051 15501092 10.1016/j.neuroimage.2004.07.051

[73] Bzdok D, Thomas Yeo BT. The Future of Data Analysis in the Neurosciences [Internet]. arXiv [q-bio.NC]. 2016 Available: http://arxiv.org/abs/1608.03465

[74] Walt S van der, Colbert SC, Varoquaux G. The NumPy array: a structure for efficient numerical computation. Comput Sci Eng. IEEE; 2011;13: 22–30.

[75] Jones E, Oliphant T, Peterson P. {SciPy}: Open source scientific tools for {Python} [Internet]. 2001—. Available: http://www.scipy.org

[76] McKinney W. pandas: a foundational Python library for data analysis and statistics. Python for High Performance and Scientific Computing. 2011; 1–9.

