# Supplementary material for: Decoding brain activity using a large-scale probabilistic functional-anatomical atlas of human cognition
Source: PLoS Comput Biol. 2017 Oct 23;13(10):e1005649. doi: 10.1371/journal.pcbi.1005649 (PMC5683652; doi:10.1371/journal.pcbi.1005649)
Supplement: S1 Supporting Results — (PDF) [file pcbi.1005649.s001.pdf]

Supporting Results

Topic-based reconstruction of whole-brain maps

The probabilistic functional-anatomical atlas we introduce in the main text is useful not only for advancing theoretical understanding of the functional properties of different brain regions, but also for facilitating description and interpretation of novel whole-brain maps. One can conceptualize the topics produced by the GC-LDA model as a basis set that can be combined in various ways to produce much more complex patterns of whole-brain activity. In principle, a diverse array of whole-brain activation maps might be recaptured or reconstructed using a weighted combination of our regionally-circumscribed topics. This capability would provide a powerful means of reducing brain maps that nominally contain hundreds of thousands of distinct voxels to a much smaller number of meaningful, functionally distinctive units.

To test this idea empirically, we attempted to "reconstruct" a series of whole-brain activation maps by fitting a regression model that predicted activation at each voxel in each target image from activation levels in the 200 region-specific topic maps. We used a regression approach to reconstruct whole-brain activation maps using the topics generated by our GC-LDA model. First, we smoothed each of the 200 topic maps containing activation assignments (e.g., Figure 3b-c) with a 6 mm FWHM kernel. Next, we vectorized both the target whole-brain map and the 200 smoothed topic maps. We then fit an ordinary least squares regression model predicting the target whole-brain activation map from the 200 topic maps, i.e.,

$$y = \alpha + \sum_{i=1}^{T} \beta_i X_i$$

where y is the vectorized input image to decode, α is the intercept, T is the number of topics, β_i is the estimated coefficient for the ith topic, and X_i is the ith smoothed whole-brain topic map

(cf. Figure 3). This analysis produces a set of 200 β coefficients that reflect the relative weight of each topic in the reconstructed/predicted map. We report the full model's coefficient of determination (R^2) as a metric of the model's relative ability to describe the original map.

We applied this reconstruction approach to three very different sets of whole-brain images, including (1) the 20 BrainMap ICA maps reported by Smith et al. [1]; (2) a randomly-selected set of 100 maps drawn from the NeuroVault whole-brain image repository [2]; and (3) single-subject activation maps from the the Human Connectome Project [HCP; 3]---a landmark study that to date has released fMRI data from over 900 subjects performing a variety of experimental tasks [4]. Supporting Figure S4 illustrates reconstruction results for sample images of each type (for additional examples, see Supporting Figures S5 - S7). For the BrainMap ICA images, reconstruction fidelity was almost universally high (mean R^2 = 0.74), and visual inspection revealed striking similarity in the vast majority of cases (Supp. Fig. 4A, Supp. Fig. 5; the sole exception was component 19 [R^2 = .23], which clearly consisted of artifactual activation on the fringe of the brain). For the NeuroVault maps---which varied widely in terms of task, analysis type, and sample size---reconstruction fidelity was somewhat lower (mean R^2 = 0.46; Supp. Figure 4B), but the reconstructed maps preserved most of the spatial detail in the original maps (Supp. Fig. 7). As a general rule, maps sourced from clearly-defined group-level contrasts were easier to reconstruct than maps with ambiguous provenance.

In contrast, reconstruction accuracy was relatively poor for the single-subject HCP images, with mean R^2 values ranging from 0.18 (social cognition task) to R^2 = 0.3 (gambling task) across 4 different HCP tasks. This decrease was expected, however, as single-subject maps are necessarily noisier than group-averaged estimates, and also reflect considerable idiosyncracies in individual anatomy. Standard mass univariate group-level analyses are

typically blind to such fine-grained differences, and can retain only the coarse patterns observed across the sample. The topic reconstruction approach can be viewed as an analogous means of regularizing low-level anatomical idiosyncracies and abstracting away high-level commonalities. That is, subjects whose neural responses to the same stimulus look very different in the original voxel space will typically have considerably more similar representations when reconstructed using our topics. For example, it is not at all apparent that the two single-subject images presented in Supp. Fig. 4C reflect the same functional task (i.e., the HCP Emotion task). By contrast, the topic-reconstructed images look much more similar, while still correlating strongly with each of the original two images.

References

1. Smith SM, Fox PT, Miller KL, Glahn DC, Fox PM, Mackay CE, et al. Correspondence of the brain’s functional architecture during activation and rest. Proc Natl Acad Sci U S A. National Academy of Sciences; 2009;106: 13040–13045.

2. Gorgolewski KJ, Varoquaux G, Rivera G, Schwarz Y, Ghosh SS, Maumet C, et al. NeuroVault.org: a web-based repository for collecting and sharing unthresholded statistical maps of the human brain. Front Neuroinform. 2015;9: 8.

3. Van Essen DC, Smith SM, Barch DM, Behrens TEJ, Yacoub E, Ugurbil K, et al. The WU-Minn Human Connectome Project: an overview. Neuroimage. 2013;80: 62–79.

4. Barch DM, Burgess GC, Harms MP, Petersen SE, Schlaggar BL, Corbetta M, et al. Function in the human connectome: task-fMRI and individual differences in behavior. Neuroimage. 2013;80: 169–189.
