# Supplementary figures and images for: Decoding brain activity using a large-scale probabilistic functional-anatomical atlas of human cognition
Source: PLoS Comput Biol. 2017 Oct 23;13(10):e1005649. doi: 10.1371/journal.pcbi.1005649 (PMC5683652; doi:10.1371/journal.pcbi.1005649)

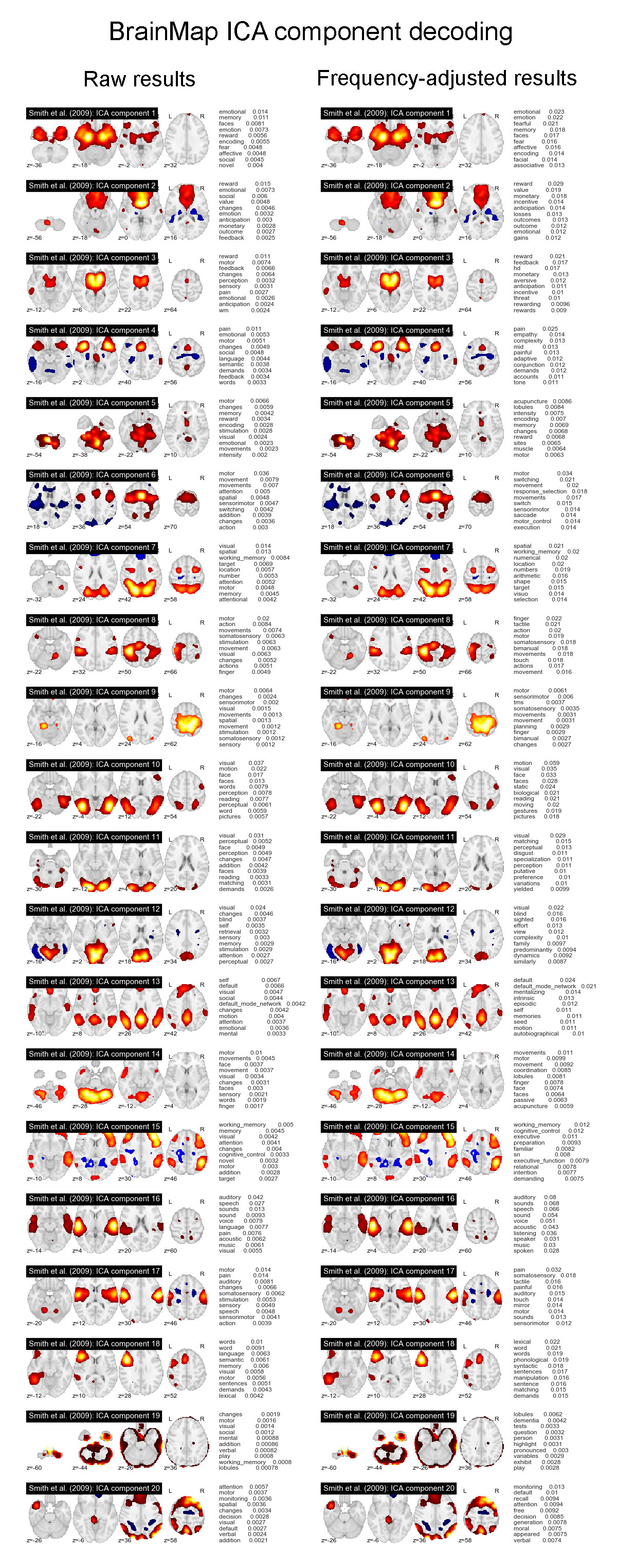

Supplement: S2 Fig — (JPG) [file pcbi.1005649.s003.jpg]

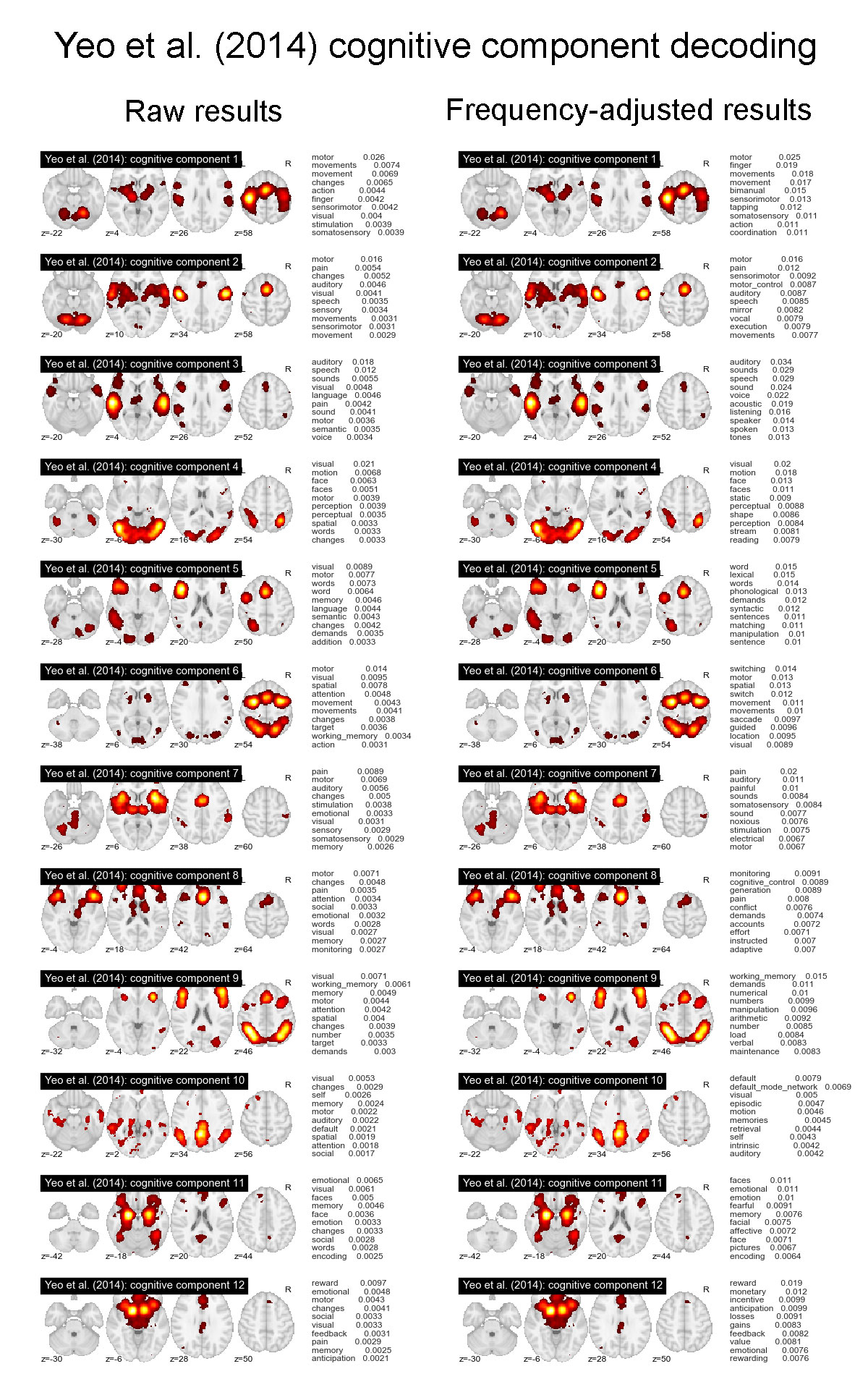

Supplement: S3 Fig — (JPG) [file pcbi.1005649.s004.jpg]

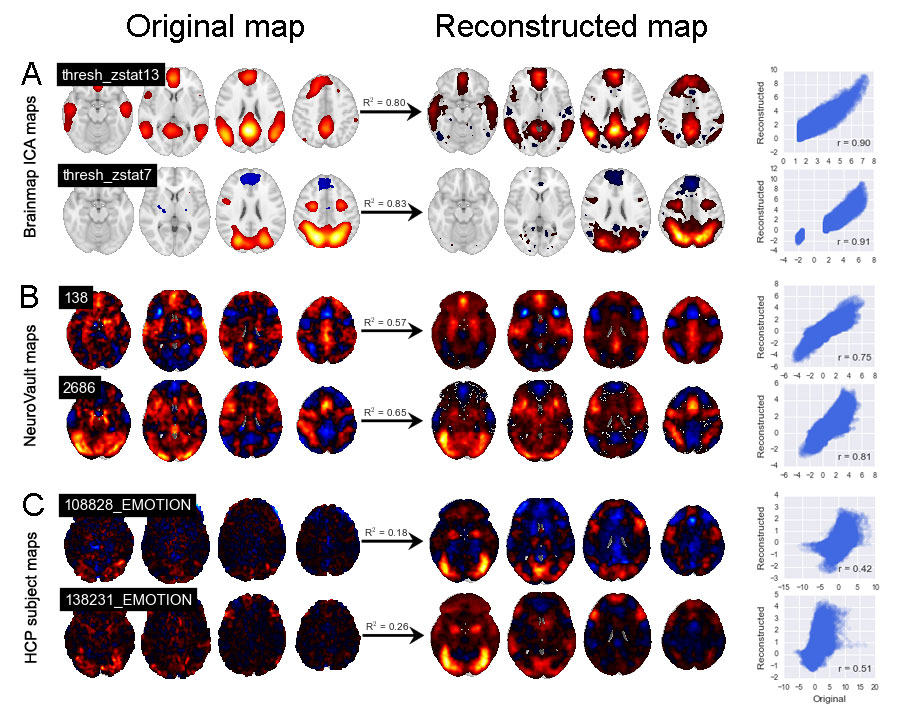

Supplement: S4 Fig — Topic-based reconstruction of whole-brain activity maps. Representative examples from (A) the set of 20 BrainMap ICA components reported in Smith et al. [15]. (B) the NeuroVault whole-brain image repository [2], and (C) single-subject contrast maps from the emotion processing task in the Human Connectome Project dataset (face vs. shape contrast). Each row displays the original (left) and reconstructed (center) image, along with the coefficient of determination (R2) for the fitted reconstruction model, and a scatter plot of all voxels (right). (JPG) [file pcbi.1005649.s005.jpg]

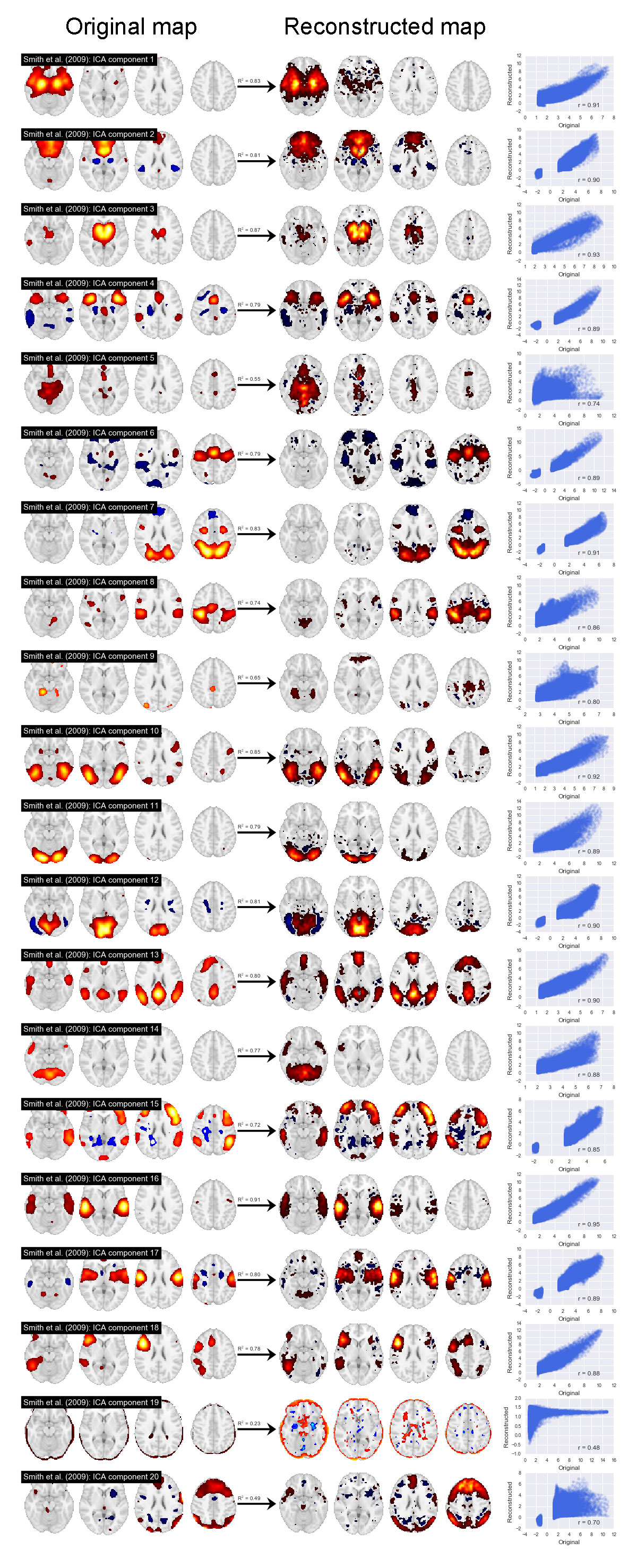

Supplement: S5 Fig — (JPG) [file pcbi.1005649.s006.jpg]

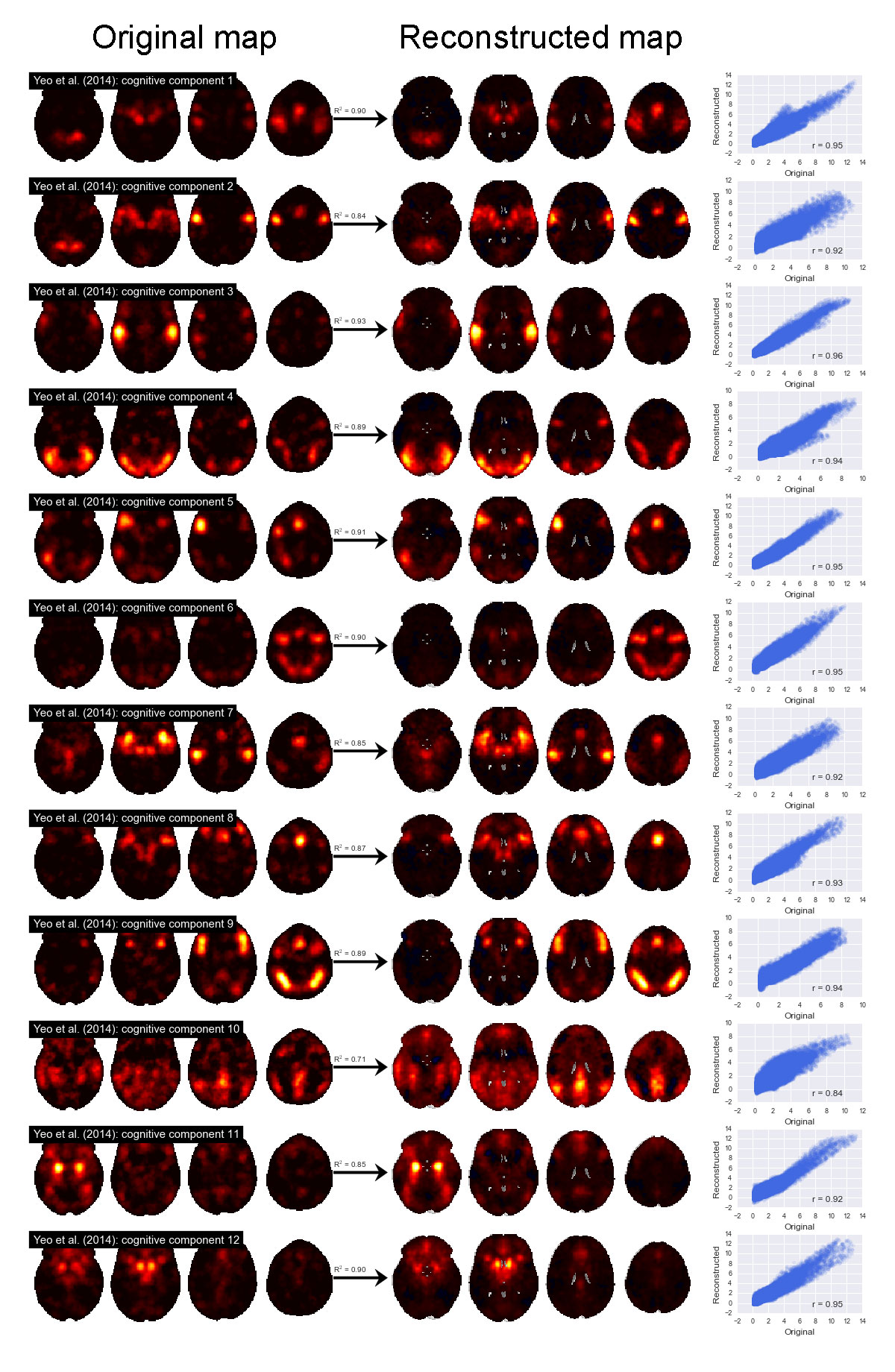

Supplement: S6 Fig — (JPG) [file pcbi.1005649.s007.jpg]

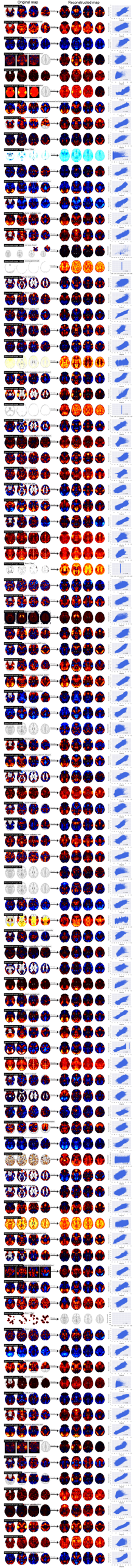

Supplement: S7 Fig — Labels in white indicate human-annotated cognitive atlas paradigm, when available. (JPG) [file pcbi.1005649.s008.jpg]
